# Monoamine oxidase and transaminase screening: biotransformation of 2-methyl-6-alkylpiperidines by *Neopestalotiopsis* sp. CBMAI 2030

**DOI:** 10.1007/s00253-017-8389-z

**Published:** 2017-06-28

**Authors:** Jonas Henrique Costa, Bruna Zucoloto da Costa, Derlene Attili de Angelis, Anita Jocelyne Marsaioli

**Affiliations:** 1 Institute of Chemistry, State University of Campinas—UNICAMP, PO Box 6154, Campinas, SP 13083-970 Brazil; 2 Division of Microbial Resources, Chemical, Biological and Agricultural Pluridisciplinary Research Center—CPQBA, State University of Campinas—UNICAMP, Campinas, SP 13148-218 Brazil

**Keywords:** Chiral amines, Fluorogenic probes, High-throughput screening, Monoamine oxidase, Piperidines, Transaminase

## Abstract

High-throughput screening detected transaminases (TAs) and monoamine oxidases (MAOs) in fungi by applying a fluorogenic probe. Strains F026, F037, F041, F053, and F057 showed the highest enzymatic conversions (31, 60, 30, 40, and 32%, respectively) and where evaluated for their ability to transform piperidines. Strain F053 (*Neopestalotiopsis* sp. CBMAI 2030) revealed unusual enzymatic activity to deracemize 2-methyl-6-alkylpiperidines. *Neopestalotiopsis* sp. CBMAI 2030 was capable to convert 2-methyl-6-propylpiperidine, 2-methyl-6-butylpiperidine, and 2-methyl-6-pentylpiperidine in piperideine with 11, 14, and 24% conversion, respectively. The activity was enhanced by cultivating the fungus with 2-methyl-6-pentylpiperidine (38% conversion and 73% *ee*).

**Electronic supplementary material:**

The online version of this article (doi:10.1007/s00253-017-8389-z) contains supplementary material, which is available to authorized users.

## Introduction

Chiral amines are often biologically active and synthetically demanding chiral intermediates with applications in pharmaceutical and agrochemical industries (Koszelewski et al. 2010; Carr et al. 2003). Chiral 2-methyl-6-alkylpiperidine moieties are present in solenopsin alkaloids, which are the main components in *Solenopsis* ant venom. These compounds usually display an (2*R*,6*S*) absolute configuration (Pianaro et al. 2012), and their proposed biosynthetic pathway suggests the participation of transaminases (TAs) and imino reductases (IREDs) in the definition of the chiral centers (Leclercq et al. 1996). Nowadays, these stereo-controlled processes can be reproduced *in lab* by using the same enzyme families (TA-IRED cascade) and diketones as substrates (France et al. 2016). Additionally, a similar cascade can also be performed by monoamine oxidases from *Aspergillus niger* (MAO-N) and ω-transaminases (TA) (Reilly et al. 2014). These enzymes are cofactor-dependent; TAs depend on pyridoxal 5′-phosphate (PLP) and MAO-N on flavin mononucleotide (FMN). TAs catalyze transamination, transferring amino groups from amines or amino acids to amine acceptors, ketones, or α-ketoacids, and are relevant to amino acid production in microorganisms and animals (Koszelewski et al. 2010; Hwang and Kim 2004). MAOs have been detected in many organisms (Atkin et al. 2008a, b) and are responsible for the oxidative deamination of monoamines, resulting in hydrogen peroxide and imine formation to produce ketones or aldehydes (Atkin et al. 2008a, b).

In microorganisms, these enzymatic activities can be detected by applying high-throughput screening (HTS) assays. Most HTS methodologies detect signals from a fluorogenic or chromogenic probe in 96-well microplates, which reveal that the enzymatic reaction occurred (Reymond 2008). Assays detecting fluorescent signals are more sensitive and provide a linear response to the reaction progress without interference of colored products (Reymond 2006).

Here, we used HTS and a fluorogenic probe recently suggested by Lu et al. (2008) to monitor MAO and/or TA enzymes in fungi available from LaBioChem *in house* culture collection, UNICAMP. The enzymatic activity of the best strain was validated in the deracemization of 2,6-dialkylpiperidines.

## Material and methods

### General methods

Commercially available reagents and solvents were acquired from Sigma-Aldrich, Acros, or Synth, and purified following standard procedures (Perrin et al. 1980) when necessary. Merck silica gel 60 (230–400 mesh ASTM) was used for “flash” column chromatography, and thin-layer chromatography was performed using Merck silica gel 60 F_254_ on aluminum foils, revealed by UV_254 nm_ lamp irradiation. Visualization spray reagents for TLC were either 10% *w*/*v* phosphomolybdic acid in ethanol or *p*-anisaldehyde, H_2_SO_4_, acetic acid in ethanol (1:2:1:100 *v*/*v*), or Dragendorff’s solution containing bismuth(III)nitrate (0.85 g), tartaric acid (10 g), and potassium iodide (16 g) in distilled water (80 mL). ^1^H NMR (600.17 MHz) and ^13^C NMR (150.91 MHz) were acquired with a Bruker Avance III 600 (*B*_0_ = 14.1 T), and ^1^H NMR (400.13 MHz) and ^13^C NMR (100.63 MHz) were acquired with a Bruker Avance III 400 (*B*_0_ = 9.4 T). Deuterated chloroform (CDCl_3_; 7.23 ppm), deuterated methanol (CD_3_OD; 3.35 and 4.78 ppm), and tetramethylsilane (TMS; 0.0 ppm) were used as a solvent and internal reference. Chemical shifts were expressed in δ (ppm) and the coupling constants (*J*) in Hertz (Hz). The GC-MS analyses were performed in an Agilent 6890 Series chromatograph coupled to a Hewlett-Packard 5973 mass spectrometer with an electron ionization source (EI) operating at 70 eV, and equipped with a fused silica capillary column HP 5-MS (30 m × 0.25 mm × 0.25 μm) with 5% phenylmethylsiloxane. Helium was used as carrier gas (1 mL min^−1^), and the injector temperature was set to 250 °C, detector temperature to 230 °C, and an injection volume of 1.0 μL in splitless mode was used. The initial oven temperature was 50 °C, increasing at 20 °C min^−1^ to 290 °C. The diastereomeric discrimination of compound **11** was achieved using an Agilent 6850 chromatograph equipped with a flame ionization detector and a fused silica capillary column Chrompack® with chiral phase Chirasil-β-cyclodextrin (25 m × 0.25 mm × 0.25 μm), H_2_ carrier gas (2 mL min^−1^), injector at 180 °C, detector at 150 °C, and splitless mode injections of 1.0 μL. Mass spectrometry with electrospray ionization (ESI-MS) was performed using a Waters Quattro Micro TM API spectrometer. Samples of compounds **1** and **1d** (10 μg/mL in methanol) were applied by direct infusion using a 50 μL min^−1^, and a 0.1 mL min^−1^ flux of 0.1% *v*/*v* formic acid in methanol was used. Nitrogen was the nebulizing gas, the analyses were performed in the positive mode, and the parameters were as follow: capillary voltage 3 kV, cone voltage 25 V, extractor voltage 3 V, RF lens voltage 0.5 V, source temperature 150 °C, desolvation temperature 200 °C, desolving gas flux 800 L/h, and gas flux at the cone 50 L/h. Hydrogenations were performed using a Parr 3926 shaker hydrogenation apparatus.

### Microorganisms

Microorganisms (MOs) from our private collection were used in HTS stage, and the hit F053 was identified as *Neopestalotiopsis* sp. and deposited at CBMAI (Brazilian Collection of Microorganisms from the Environment and Industry) under the code name CBMAI 2030. For the HTS assays, the MOs were inoculated on Petri dishes containing malt extract agar (MEA) culture medium and incubated for 72 h at 30 °C. The cells were suspended in 20 mM borate buffer pH 7.4 to a final concentration of 1 mg mL^−1^.

For the biotransformations, MOs were transferred to an Erlenmeyer flask (500 mL) containing 200 mL of malt extract (ME) culture medium and incubated at 30 °C and 200 rpm for 48 h. The cells were harvested by filtration under vacuum and used direct in the assays.

### HTS assays

Screening with whole cells were performed using 7-(3-aminopropoxy)coumarin (probe **1**) as fluorogenic substrate and following a previously optimized protocol described by Bicalho et al. (2004). The HTS assays were developed in 96-well polypropylene microtiter plates in quadruplicate, and reaction controls in duplicate.

- Assay (A): 10 μL of 2 mM probe **1** solution in water:acetonitrile (1:1 *v*/*v*), 80 μL of 5.0 mg mL^−1^ BSA in 20 mM borate buffer pH 7.4, 10 μL of 20 mM borate buffer pH 7.4, and 100 μL of 1.0 mg mL^−1^ cell suspension in 20 mM borate buffer pH 7.4.
- Negative control (NC): 10 μL of 2 mM probe **1** solution in water:acetonitrile (1:1 *v*/*v*), 80 μL of 5.0 mg mL^−1^ BSA solution in 20 mM borate buffer pH 7.4, and 110 μL of 20 mM borate buffer pH 7.4.
- Positive control (PC): 10 μL of 2 mM umbelliferone solution in water:acetonitrile (1:1 *v*/*v*), 80 μL of 5.0 mg mL^−1^ BSA solution in 20 mM borate buffer pH 7.4, 10 μL of 20 mM borate buffer pH 7.4, and 100 μL of 1.0 mg mL^−1^ cell suspension in 20 mM borate buffer pH 7.4.

The microtiter plates were incubated at 30 °C and 200 rpm. The fluorescent signal was monitored using a PerkinElmer EnSpire microtiter plate reader at 0, 24, 48, 72, and 96 h (excitation wavelength *λ*_ex_ = 360 nm and emission wavelength *λ*_em_ = 460 nm).

The results were expressed as percentage of conversion (%) applying Eq. 1. All results were averaged and conversion values are estimated, taking the positive control as 100%.

1 $$ \%=\frac{\left( A- NC\right)}{PC}\times 100 $$

### Biotransformation reactions with 2-methyl-6-alkylpiperidines

To an Erlenmeyer flask (50 mL) containing 1 g of wet cells in 20 mL of 100 mM Sørensen’s phosphate buffer pH 7.0 (Sørensen 1909), compounds **6**, **7**, or **8** (10 mg) was added. The resulting mixture was incubated at 200 rpm and 30 °C. Biotransformation reactions and negative controls were monitored as follow: 1-mL aliquots were saturated with NaCl, basified with 5.0 M NaOH (100 μL), extracted with ethyl acetate (2 × 0.5 mL), and centrifuged at 14,000 rpm; the organic layers were combined and analyzed by GC-MS, adding benzophenone (0.1 mg mL^−1^) as internal standard.

### Evolution experiments of *Neopestalotiopsis* sp. CBMAI 2030

Erlenmeyer flasks (500 mL) containing compound **8** (10 mg), ME culture medium (200 mL), and *Neopestalotiopsis* sp. CBMAI 2030 cells were incubated at 200 rpm and 30 °C for 48 h. The cells were filtered, washed with 100 mM Sørensen buffer pH 7.0, and added to the biotransformation reactions. After 15 days, the cells were harvested and added to a new ME culture medium containing compound **8** (10 mg). This procedure was repeated three times.

### Synthetic procedures

#### *Tert*-butyl-3-chloropropylcarbamate (1c)

Triethylamine (1.67 mL, 12 mmol) was added dropwise to a solution of 3-chloropropylamine (**1a**, 1.30 g, 10 mmol) in dichloromethane (4.5 mL) in a two-neck round-bottom flask. Then, a di-*tert-*butyldicarbonate (**1b**) (2.18 g, 10 mmol) solution in dichloromethane (4.5 mL) was added dropwise to the reaction mixture and stirred at room temperature for 18 h. The reaction was quenched by addition of dichloromethane (10 mL), and the organic phase was washed with 1 M HCl (1 × 10 mL), water (2 × 5 mL), NaHCO_3_ saturated solution (1 × 10 mL), and brine (1 × 10 mL). The organic layer was dried over anhydrous MgSO_4_ and the solvent was evaporated under reduced pressure. Compound **1c** was obtained as an oily residue (1.60 g) in 83% yield. No further purification step was necessary.

M.W.: 193.6 g mol^−1^ (C_8_H_16_ClNO_2_). ^**1**^ **H NMR** (400.18 MHz, CDCl_3_, δ_TMS_ 0.00) δ 3.59 (2H, t, *J* = 6 Hz, H-1), 3.28 (2H, q, *J* = 6 Hz, H-3), 1.97 (2H, quint, *J* = 6 Hz, H-2), 1.44 (9H, s, H-6, H-7, H-8). ^**13**^ **C NMR** (100.63 MHz, CDCl_3_, δ_CDCl3_ 77.0): δ 155.9 (C, C-4), 79.4 (C, C-5), 42.3 (CH_2_, C-1), 37.9 (CH_2_, C-3), 32.6 (CH_2_, C-2), 28.4 (CH_3_, C-6, C-7, C-8). **EI-MS (70 eV)** *m/z* (%): 193 (M^•+^, not present), 140 (14), 138 (41), 137 (4), 134 (5), 94 (5), 93 (4), 59 (48), 58 (7), 57 (100), 56 (11). **TLC** (ethyl acetate, phosphomolybdic acid stain solution) R_ƒ_ = 0.6. **Spectra:** See Figs. S1, S2, and S3 in Online Resource 1.

#### *Tert*-butyl (3-((2-oxo-2*H*-chromen-7-yloxy)propyl)carbamate (1d)

Umbelliferone (0.325 g, 2 mmol) and acetone (10 mL) were added to a two-neck round-bottom flask under nitrogen atmosphere. The mixture was cooled to 0 °C and **1c** (0.956 g, 5 mmol); potassium carbonate (0.47 g, 3.5 mmol) and sodium iodide (0.45 g, 3.0 mmol) were added. The reaction was kept under reflux for 18 h. The solvent was evaporated under reduced pressure and the crude residue was purified by silica gel column chromatography eluted with hexane:ethyl acetate (2:1 *v*/*v*) to produce 0.57 g of solid **1d**, in 89% yield.

M.W.: 319.1 g mol^−1^ (C_17_H_21_NO_5_). ^**1**^ **H NMR** (400.18 MHz, CD_3_OD, δ_CD3OD_ 4.87 ppm): δ 7.86 (1H, d, *J* = 9 Hz, H-3), 7.51 (1H, d, *J* = 9 Hz, H-9), 6.92 (1H, dd, *J* = 9 e 2 Hz, H-8), 6.88 (1H, d, *J* = 2 Hz, H-6), 6.23 (1H, d, *J* = 9 Hz, H-2), 4.10 (2H, t, *J* = 6 Hz, H-10), 3.24 (2H, q, *J* = 6 Hz, H-12), 1.97 (2H, quint, *J* = 6 Hz, H-11), 1.42 (9H, s, H-15,H-16, H-17). ^**13**^ **C NMR** (100.63 MHz, CD_3_OD, δ_CD3OD_ 49,2 ppm): δ 164.1 (C, C-7), 163.5 (C, C-1), 158.7 (C, C-13), 157.2 (C, C-5), 145.9 (CH, C-3), 130.6 (CH, C-9), 114.4 (CH, C-8), 114.1 (C, C-4), 113.5 (CH, C-2), 102.4 (CH, C-6), 80.1 (C, C-14), 67.5 (CH_2_, C-10), 38.4 (CH_2_, C-12), 30.7 (CH_2_, C-11), 28.9 (CH_3_, C-15, C-16, C17). **ESI** ^**+**^ **/MS**: 358.1 (C_17_H_20_NO_5_K^+^), 342.1 (C_17_H_20_NO_5_Na^+^), 320.1 (C_17_H_21_NO_5_H^+^). **TLC** (hexane:ethyl acetate, 1:1 *v*/*v*, phosphomolybdic acid stain solution) R_ƒ_ = 0.4. **Spectra:** See Figs. S4, S5, and S6 in Online Resource 1.

#### 7-(3-Aminopropoxy)coumarin (1)

Compound **1d** (160 mg, 0.5 mmol), water:trifluoroacetic acid 1:1 *v*/*v* (10 mL), dichloromethane (10 mL), and triisopropylsilane (20 μL) were added to a two-neck round-bottom flask. The mixture was stirred at room temperature for 30 min. The crude product was purified using a Sep-Pak column (C18, 3 cm^3^, Waters) and eluted with methanol. Compound **1** was obtained as a whitish solid in quantitative yield (0.125 g).

M.W.: 219.2 g mol^−1^ (C_12_H_13_NO_3_). ^**1**^ **H NMR** (600 MHz, CD_3_OD, δ_CD3OD_ 4.87 ppm): δ 7.91 (1H, d, *J* = 9 Hz, H-3), 7.58 (1H, d, *J* = 9 Hz, H-9), 6.98 (1H, dd, *J* = 9 e 2.4 Hz, H-8), 6.96 (1H, d, *J* = 2.4 Hz, H-6), 6.28 (1H, d, J = 9 Hz, H-2), 4.23 (2H, t, *J* = 6 Hz, H-10), 3.19 (2H, q, *J* = 6 Hz, H-12), 2.20 (2H, quint, *J* = 6 Hz, H-11). ^**13**^ **C NMR** (150 MHz, CD_3_OD, δ_CD3OD_ 49.2 ppm): δ 163.5 (C, C-7), 163.4 (C, C-1), 157.2 (C, C-5), 145.8 (CH, C-3), 130.7 (CH, C-9), 114.7 (C, C-4), 114.2 (CH, C-8), 113.9 (CH, C-2), 102.6 (CH, C-6), 67.0 (CH_2_, C-10), 38.6 (CH_2_, C-12), δ 28.3 (CH_2_, C-11). **ESI** ^**+**^ **/MS**: 220.3 (C_12_H_13_NO_3_H^+^). **TLC** (hexane:ethyl acetate, 1:1 *v*/*v*, phosphomolybdic acid stain solution) *R*_*ƒ*_ = 0.2. **Spectra:** See Figs. S7, S8, and S9 in Online Resource 1.

#### 2-Methyl-6-alkylpyridine (6b, 7b, and 8b)

To a stirring solution of 2,6-lutidine (**9**) (2 mL) in dry THF (15 mL), under nitrogen atmosphere and at 0 °C, 1.6 M BuLi in hexane (*n-*butyl lithium, 13 mL) was added slowly. The reaction was maintained at room temperature for 15 min and then heated to reflux for 15 min. The reaction mixture was cooled to 0 °C, and iodoethane (1.65 mL), allylbromide (1.79 mL), or 1-bromobutane (2 mL) was slowly added. The reaction was stirred for 18 h. Work-up was performed with cold water (25 mL) and by extraction with ethyl acetate (2 × 20 mL). The organic layers were combined and dried over anhydrous MgSO_4_. The solvent was evaporated and the crude product was purified by silica gel column chromatography (hexane:ethyl acetate, 9:1 *v*/*v*) to yield 1.41, 1.65, and 1.70 g of **6b**, **7b**, and **8b**, respectively, as yellow oils in 60, 64, and 60% yield, respectively.

2-methyl-6-propylpyridine (**6b**) M.W.: 135.1 g mol^−1^ (C_9_H_13_N). ^**1**^ **H NMR** (600.17 MHz, CDCl_3_, δ_TMS_ 0.00): δ 7.47 (1H, t, *J* = 7.8 Hz, H-4), 6.95 (2H, t, *J* = 7.8 Hz, H-3, H-5), 2.73 (2H, t, *J* = 7,8 Hz, H-7), 2.53 (3H, s, H-10), 1.70 (2H, sex, *J* = 7,8 Hz, H-8), 0.97 (3H, t, J = 7,8 Hz, H-9). ^**13**^ **C NMR** (150.92 MHz, CDCl_3_, δ_CDCl3_ 77,0): δ 161.9 (C, C-2), 157.9 (C, C-6), 136.6 (CH, C-4), 120.6 (CH, C-3), 119.7 (CH, C-5), 40.7 (CH_2_, C-7), 24.7 (CH_3_, C-10), 23.6 (CH_2_, C-8), 14.1 (CH_2_, C-9). **EI/MS (70 eV)** *m/z* (%): 135 (M^•+^, 3), 134 (15), 120 (32), 108 (9), 107 (100), 106 (7), 93 (5), 92 (5), 77 (5), 65 (4), 66 (6). **Spectra:** See Figs. S10, S11, and S12 in Online Resource 1.

2-methyl-6-(but-3-en-1-yl)pyridine (**7b**) M.W.: 147.1 g mol^−1^ (C_10_H_13_N). ^**1**^ **H NMR** (400.18 MHz, CDCl_3_, δ_TMS_ 0.00): δ 7.47 (1H, t, *J* = 7.6 Hz, H-4), 6.95 (2H, t, *J* = 6.8 Hz, H-3, H-5), 5.87 (1H, m, J = 17.2, 10 e 6.4 Hz, H-9), 5.05 (1H, d, *J* = 16.8 Hz, H-10 *trans*), 4.97 (1H, d, *J* = 10.0 Hz, H-10 *cis*), 2.85 (2H, t, *J* = 7.6 Hz, H-7), 2.53 (3H, s, H-11), 2.47 (2H, q, *J* = 7.2 Hz, H-8). ^**13**^ **C NMR** (100.63 MHz, CDCl_3_, δ_CDCl3_ 77,0): δ 161.0 (C, C-2), 157.9 (C, C-6), 138.1 (CH, C-9), 136.7 (CH, C-4), 120.7 (CH, C-3), 119.8 (CH, C-5), 115.1(CH_2_, C-10), 37.9 (CH_2_, C-7), 34.2 (CH_2_, C-8), 24.7 (CH_3_, C-11). **IE/EM (70 eV)** *m*/*z* (%): 147 (M ^•+^, 57), 146 (100), 144 (9), 132 (47), 131 (35), 130 (9), 107 (25), 106 (7), 93 (14), 77 (11). **Spectra:** See Figs. S13, S14, and S15 in Online Resource 1.

2-methyl-6-pentylpyridine (**8b**) M.W.: 163.2 g mol^−1^ (C_11_H_17_N). ^**1**^ **H NMR** (400.18 MHz, CDCl_3_, δ_TMS_ 0,00): δ 7.47 (1H, t, *J* = 7.6 Hz, H-4), 6.95 (2H, dd, *J* = 3.3 and 7.6 Hz, H-3, H-5), 2.74 (2H, t, *J* = 7.8 Hz, H-7), 2.53 (3H, s, H-12), 1,70 (2H, quin, *J* = 5.3 Hz, H-8), 1.35 (4H, m, H-9, H-10), 0.89 (3H, t, J = 6.9 Hz, H-11). ^**13**^ **C NMR** (100.63 MHz, CDCl_3_, δ_CDCl3_ 77.0): δ 161.9 (C, C-2), 157.6 (C, C-6), 136.4 (CH, C-4), 120.3 (CH, C-3), 119.4 (CH, C-5), 38.6 (CH_2_, C-7), 31.7 (CH_2_, C-8), 29.9 (CH_2_, C-9), 24.5 (CH_3_, C-12), 22.5 (CH_2_, C-10), 14.0 (CH_2_, C-11). **EI/MS (70 eV)** *m*/*z* (%): 163 (M ^•+^, 1), 134 (28), 132 (3), 121 (6), 120 (27), 108 (9), 107 (100), 106 (7), 92 (4), 77 (5), 65 (4). **Spectra:** See Figs. S16, S17, and S18 in Online Resource 1. **TLC **(hexane:ethyl acetate, 8:2 v/v, anisaldehyde stain solution) Rf = 0.6.

#### 2-Methyl-6-alkylpiperidines (6, 7, and 8)

Solutions of **6b**, **7b**, or **8b** (0.5 g) in methanol (8 mL) and glacial acetic acid (40 mL) with Pt/C 10% (100 mg) were hydrogenated at 60 bar H_2_ and 500 rpm for 48 h using a Parr 3926 shaker hydrogenator. The reaction mixture was filtered over a Celite 545 pad (2 g) and eluted with methanol (50 mL). Solvent evaporation yielded the crude product, which was purified by silica gel column chromatography, eluted with methanol, to yield 0.5, 0.48, and 0.5 g of **6**, **7**, and **8**, respectively, as yellow oils in 99, 98, and 99% yield, respectively.

2-methyl-6-propylpiperidine (**6**) M.W.: 141.2 g mol^−1^ (C_9_H_19_N). ^**1**^ **H NMR** (600.18 MHz, CDCl_3_, δ_TMS_ 0.00): δ 3.11 (1H, m, H-2), 2.96 (1H, s, H-6), 1.90–1.25 (14H, m), 0.92 (3H, t, *J* = 11 Hz, H-9). ^**13**^ **C NMR** (150.93 MHz, CDCl_3_, δ_CDCl3_ 77.0) δ 57.2 (CH, C-2), 53.6 (CH, C-6), 35.4 (CH_2_, C-7), 30.8 (CH_2_, C-3), 28.0 (CH_2_, C-5), 22.9 (CH_2_, C-4), 19.2 (CH_3_, C-10), 18.5 (CH_2_, C-8), 13.8 (CH_2_, C-9). **EI/MS (70 eV)** *m*/*z* (%) 141 (M^•+^, 2), 126 (8), 112 (3), 99 (8), 98 (100), 84 (3), 81 (3), 70 (5), 56 (5), 55 (5). **Spectra:** See Figs. S19, S20, and S21 in Online Resource 1.

2-methyl-6-butylpiperidine (**7**) M.W.: 155.3 g mol^−1^ (C_10_H_21_N). ^**1**^ **H NMR** (400.18 MHz, CDCl_3_, δ_TMS_ 0.00): δ 3.02 (1H, m, H-2), 2.86 (1H, s, H-6), 1.90–1,31 (16H, m), 0,88 (3H, t, *J* = 7,2 Hz, H-10).^**13**^ **C NMR** (100.63 MHz, CDCl_3_, δ_CDCl3_ 77.0): δ 57.5 (CH, C-2), 53.6 (CH, C-6), 33.3 (CH_2_, C-7), 31.4 (CH_2_, C-3), 28.7 (CH_2_, C-5), 27.7 (CH_2_, C-4), 23.3 (CH_2_, C-8), 23.1 (CH_3_, C-11), 22.5 (CH_2_, C-9), 19.6 (CH_2_, C-10). **EI/MS (70 eV)** *m*/*z* (%) 154 (M^•+^,3), 140 (7), 112 (3), 99 (7), 98 (100), 84 (3), 81 (3), 70 (5), 56 (5), 55 (5). **Spectra:** See Figs. S22, S23, and S24 in Online Resource 1.

2-methyl-6-pentylpiperidine (**8**) M.W.: 169.3 g mol^−1^ (C_11_H_23_N). ^**1**^ **H NMR** (400.18 MHz, CDCl_3_, δ_TMS_ 0,00): δ 3.05 (1H, m, H-2), 2.88 (1H, s, H-6), 2.00–1.25 (18H, m), 0.87 (3H, t, *J* = 8 Hz, H-11). ^**13**^ **C NMR** (100.63 MHz, CDCl_3_, δ_CDCl3_ 77.0): δ 57.4 (CH, C-2), 53.4 (CH, C-6), 33.3 (CH_2_, C-7), 31.4 (CH_2_, C-3), 30.8 (CH_2_, C-5), 27.9 (CH_2_, C-4), 25.0 (CH_2_, C-8), 23.0 (CH_2_, C-9), 22.8 (CH_3_, C-12), 22.5 (CH_2_, C-10), 19.2 (CH_2_, C-11). **EI/MS (70 eV)** *m*/*z* (%) 169 (M^•+^, 2), 168 (3), 154 (7), 126 (2), 99 (7), 98 (100), 81 (2), 70 (4), 69 (2), 56 (3), 55 (3). **TLC** (methanol:chloroform 5:95, Dragendorff stain solution) RF = 0.5. **Spectra:** See Figs. S25, S26, and S27 in Online Resource 1.

#### 2,2,2-Trifluor-1-(2-methyl-6-pentylpiperidine-1-il)ethanone (11)

Diethyl ether (1 mL) was used to solubilize 2-methyl-6-pentylpiperidine (**8**) (2 mg). Dry pyridine (0.8 mL) and trifluoroacetic anhydride (200 μL) were added slowly, and the reaction was kept at 30 °C for 30 min. The reaction was quenched by adding ethyl acetate (4 mL) and an aqueous solution of copper sulfate (4 × 4 mL). The organic layer was separated and dried over anhydrous MgSO_4_. The solvent was evaporated under reduced pressure to yield **11** (2.3 mg, 99%) as an oily residue.

MM: 265.3 g mol^−1^ (C_13_H_22_F_3_NO). **EI/MS (70 eV)** *m*/*z* (%): 265 (M^•+^, 1), 222 (2), 196 (4), 195 (10), 194 (100), 152 (3), 140 (9), 81 (12), 69 (4), 67 (2), 55 (15). **TLC** (hexane:dichloromethane, 8:2 *v*/*v*, anisaldehyde stain solution) *R*_*ƒ*_ = 0.7. **Spectra:** See Fig. S28 in Online Resource 1.

### Fungal identification

#### Molecular analysis

DNA extraction was performed following the protocol described by Raeder and Broda (1985). The isolate was screened for ITS loci using the ITS1 and ITS4 primers (White et al. 1990). Amplification reactions were performed using PCR with genomic DNA as a template, and the conditions were set as follows: an initial denaturation temperature of 94 °C for 2 min, 30 cycles of denaturation at 94 °C for 1 min, primer annealing at 55 °C for 1 min, primer extension at 72 °C for 3 min, and a final extension step at 72 °C for 3 min and 4 °C. Amplicons were purified using GFX PCR DNA and a Gel Band Purification Kit (GE Healthcare) and sequenced with BigDye Terminator (Life Technologies, USA) and an ABI3500XL Series Sequencer (Applied Biosystems) according to the manufacturer’s instructions. The BioEdit Sequence Alignment Editor v. 7.0.5.3 (Hall 1999) was used to generate the consensus sequence, which was compared against the GenBank nucleotide database (http://www.ncbi.nlm.nih.gov) and CBS (http://www.cbs.knaw.nl/). The most similar sequences were selected, combined with the sequence from the isolate. After alignment using the CLUSTAL X software (Thompson et al. 1994), a phylogenetic tree was generated with MEGA software version 4.0 (Tamura et al. 2007). Neighbor-joining criteria (Saitou and Nei 1987) were used in the analysis, and bootstrap values were calculated for under 1000 pseudoreplicates.

#### Morphological analysis

A single-cell preparation was used to ensure the purity of the fungal culture as follows: after a strongly diluted spore suspension was prepared in distilled water, 100 μL was transferred to a Neubauer chamber to count the number of colony forming units (CFUs). Further dilutions were performed until no more than 5 CFU was found. Then, 100 μL of the final diluted suspension was streaked onto a potato dextrose agar (PDA) layer on Petri dishes using a Drigalski spatula. The plates were incubated at 25 °C until isolated growing colonies were observed. Later, microscopic examination was based on slide preparation by adding a drop of Lactophenol Cotton Blue to a microscope slide and subsequently transferring the fungal material from the culture (Crous et al. 2009).

The ITS sequence of *Neopestalotiopsis* sp. CBMAI 2030 was deposited at GenBank with accession number KY696576.

## Results

### MAO/TA high-throughput screening

**Fig. 1:**
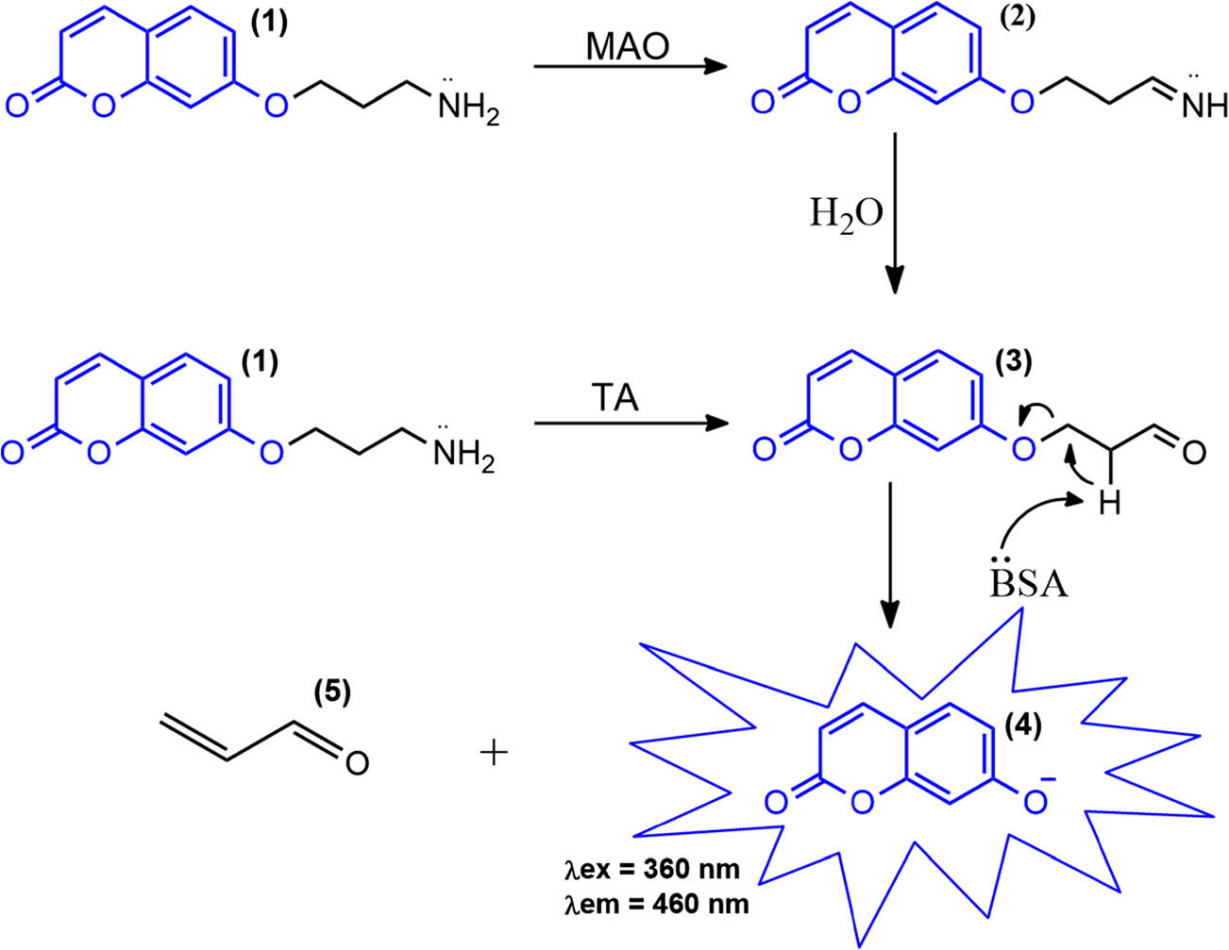
Fluorogenic assay to detect monoamine oxidases (MAOs) and transaminases (TAs). *BSA* bovine serum albumin

The fluorogenic probe 7-(3-aminopropoxy)coumarin (**1**) was synthesized in 83% yield and was successfully applied to the high-throughput screening of 39 fungi in order to quickly select the best strain for TA and/or MAO enzymatic activities (Fig. 1).

The MAO/TA HTS results revealed that 25 fungi catalyzed the oxidative deamination or transamination of probe **1** at a relatively low conversion (Table 1). On the other hand, the fluorogenic probe was converted into umbelliferone with 30–60% conversion by five fungi (Table 1): F026, F037, F041, F053, and F057. These microorganisms were selected for further investigation regarding the biotransformation of 2,6-dialkylpiperidines.

**Table 1:** Enzymatic conversion (%) of fluorogenic probe 1 by fungi

Identification number	Conversion^a^ (%)			
	24 h	48 h	72 h	96 h
F023	19	23	24	11
F024	13	17	19	13
F025	0	0	0	0
F026	18	23	27	31
F027	15	21	23	25
F028	10	15	17	20
F029	13	16	16	20
F030	1	1	2	3
F031	0.5	1	1	2
F032	6	6	10	13
F033	0.5	1	1	1
F034	2	3	4	5
F035	3	4	6	7
F037	43	60	60	60
F038	2	3	4	5
F039	6	8	9	11
F040	9	12	14	19
F041	9	13	16	30
F042	5	6	9	10
F043	0	1	1	1
F045	12	16	22	25
F046	16	18	19	22
F047	4	5	7	10
F048	0	0	1	2
F049	1	1	2	3
F050	1	2	2	4
F051	1	2	3	3
F052	1	1	2	2
F053	22	29	35	40
F054	1	1	1	1
F055	2	2	3	3
F056	2	4	6	7
F057	17	20	24	32
F058	3	4	5	6
F059	4	7	9	12
F060	1	2	3	4
F062	2	3	4	5
F063	6	14	19	21
L019	0	0	0	1

^a^Calculated using the Equation 1 presented in the experimental procedure

### Synthesis and biotransformation of 2-methyl-6-alkylpiperidines

**Fig. 2:**
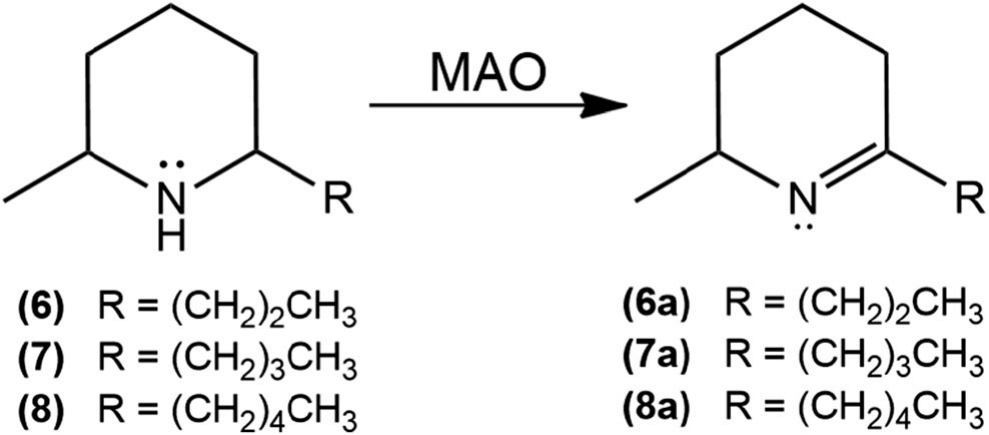
Biotransformation of 2-methyl-6-alkylpiperidines by MAO

2-Methyl-6-propylpiperidine (**6**), 2-methyl-6-butylpiperidine (**7**), and 2-methyl-6-pentylpiperidine (**8**) were synthesized, as depicted in Fig. 2, aiming at confirming possible MAO activities observed in the HTS experiments.

**Fig. 3:**
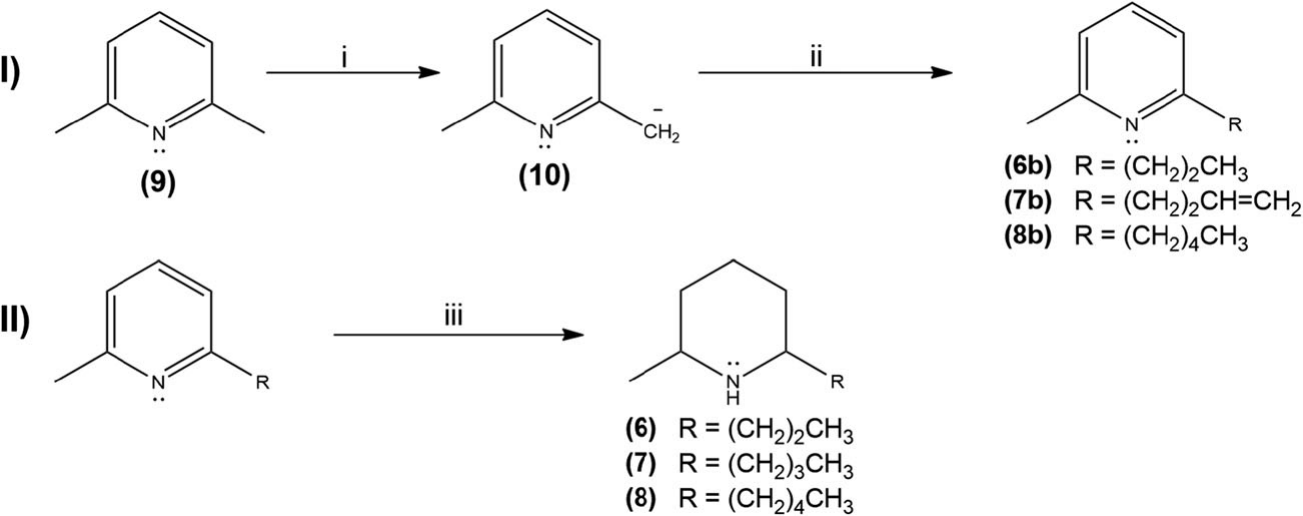
Synthetic route from 2,6-lutidine (**9**) to 2-methyl-6-alkylpiperidines. Reagents and conditions: (i) *n*-BuLi, anhydrous THF, 0 °C to r.t.; (ii) alkyl halide; and (iii) Pt/C (10%), 60 bar H_2_, CH_3_COOH/MeOH (5:1 *v*/*v*), r.t., 48 h

The synthetic route for **6**, **7**, and **8** (Fig. 3) was based on the synthesis of racemic solenopsins (Pianaro et al. 2012). The 2,6-lutidin carbanion (**10**) was obtained by treating lutidine with butyl lithium, which promptly reacted with the appropriate alkyl halide to produce 2-methyl-6-alkylpiridines **6b**, **7b**, and **8b**. In the second step, the pyridines were hydrogenated in the presence of 10% Pt/C to produce piperidines **6**, **7**, and **8**. All products and intermediates were fully characterized.

Among the five fungi selected, only F053 showed good MAO activity when tested with **6**, **7**, and **8**. The remaining microorganisms showed no activity with these piperidines (Table 2).

**Table 2:** Biotransformation of **6**, **7**, and **8**, with selected fungi

Microorganism	Conversion^a,b^ (%)		
	6	7	8
F026	–	–	–
F037	–	–	–
F041	–	–	–
F053	11	14	24
F057	–	–	–

^a^Determined by the area ratio of the product and internal standard area

^b^Final values after 14 days of reaction

**Fig. 4:**
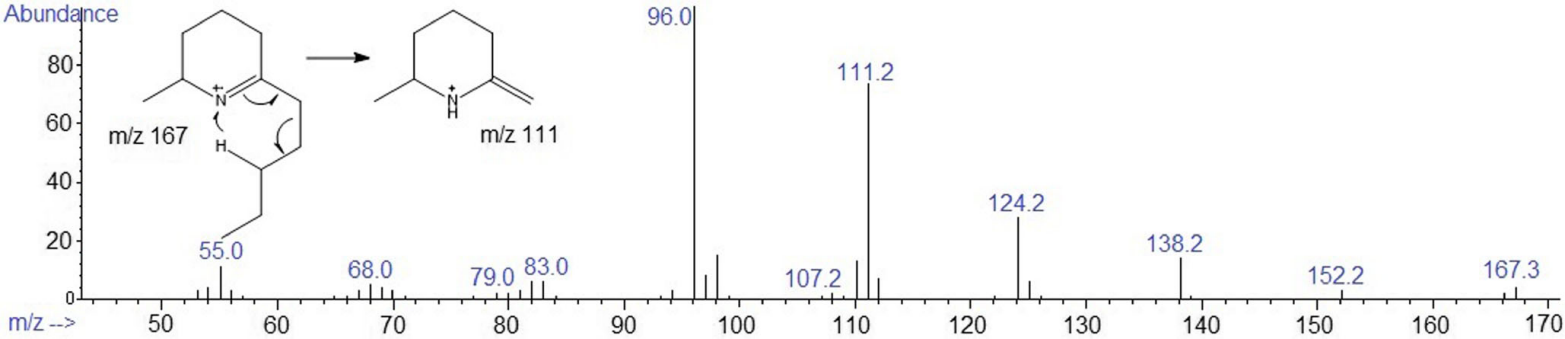
Mass spectrum EI (70 eV) for **8a**

The piperideines **6a**, **7a**, and **8a** were detected in the reaction media after 7 days with 11, 14, and 24% conversion, respectively. Using mass spectrometry, **6a**, **7a**, and **8a** were confirmed, by a characteristic fragment at *m*/*z* 111 that is rationalized by hydrogen rearrangement through a six-membered ring intermediate, as in the McLafferty rearrangement (McLafferty and Turecek 1993) (Fig. 4).

Additionally, elongation of the 2-alkyl chain length seems to increase the conversion efficiency of the piperidine into piperideine. Therefore, piperideine **8a** formation probably relies on a better acceptance of **8** by fungus F053, and this substrate was well suited for the investigation of evolutionary strategies in a laboratory setting, which is usually applied to bacteria with a short generation time. The experimental evolution of *Escherichia coli* for 40,000 generations in a glucose-limited medium is one of the best-known examples in the field (Bachmann et al. 2012). The present example is the evolution of the F053 fungus toward piperidine **8**, and each evolution cycle involved 7 to 15 days. The adaptation lasted three generations to reach a 38% of **8a**. The third generation produced **8a** in higher conversion (38%) and in shorter time (7 days). No further increase was obtained in future generations.

**Fig. 5:**
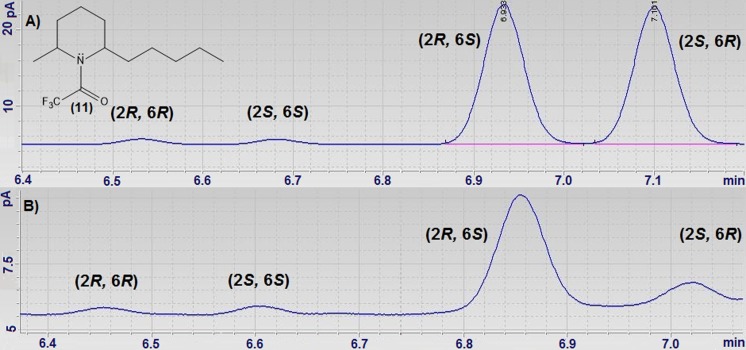
Chiral GC-FID chromatograms of **11 a** before and **b** after biotransformation of **8** by fungus F053 (*Neopestalotiopsis* sp. CBMAI 2030)

Determination of enantiomeric excess of either **8** or **8a** was not an easy task as pure **8** or **8a** was not commercially available. The strategy was to use the methodology previously applied by Pianaro et al. (2012) to reveal the absolute configuration of the solenopsins. Racemic *cis* and *trans* **8** were trifluoroacetylated (**11**) and the mixture was analyzed using chiral GC-FID with a β-cyclodextrin column revealing that **8** was mainly composed of *cis* isomers. This was confirmed by ^1^H NMR analyses, with hydrogen H-2 and H-6 chemical shifts as diagnostic (H-6 2.88 ppm and H-2 3.05 ppm) compared to those of the solenopsins (2-methyl-6-undecylpiperidines, *cis* H-6 2.85 ppm and H-2 3.03 ppm, trans H-6 3.27 ppm and H-2 3.51 ppm) (Pianaro et al. 2012). The *cis*-2-methyl-6-pentylpiperidine isomers 2*R*,6*S* (6.93 min) and 2*S*,6*R* (7.10 min) were eluted after small amounts of *trans* isomers 2*R*,6*R* (6.53 min) and 2*S*,6*S* (6.68 min). The chromatographic separation of the trifluoroacetyl derivatives of the 2-methyl-6-pentylpiperidines (**11**) on the chiral column was similar to Pianaro’s et al. (2012) (Fig. 5a). Therefore, analysis of the biotransformation of **8** by fungus F053 revealed an enantiomeric excess of 73% for the *cis* (2*S*,6*R*)-2-methyl-6-pentylpiperidine (Fig. 5b and Table 3).

**Table 3:** Biotransformation of compound **8** by *Neopestalotiopsis* sp*.* CBMAI 2030

Microorganism	Conversion^a,b^ (%)	*ee* ^c^ (%)	Selectivity
F053 (*Neopestalotiopsis* sp*.* CBMAI 2030)	38	73	(2*S*,6*R*)

^a^Average using area ratio of product and internal standard

^b^After 21 days of bioreaction with third-generation cells

^c^Enantiomeric excess, *ee* = ((*A* − *B*) / (*A* + *B*)) × 100, where *A* and *B* are the areas of the chromatogram peaks of the enantiomers

### Fungal analysis—identification

Molecular analysis of the consensus sequence of the F053 strain indicated that the isolate belongs to the genus *Neopestalotiopsis.* However, no data enabled identification at the species level. Microscopic observations on PDA culture medium revealed the presence of globose-subglobose pycnidial conidiomatas and discrete conidiogenous cells. Conidia are straight to slightly curved, 4-septate, thin-walled, versicolorous median cells. Hyaline cylindrical apical cells with tubular appendages (branched or not) and basal single, tubular, unbranched appendages were also present. Based on molecular and morphological aspects, the F053 strain was identified as *Neopestalotiopsis* sp. (Maharachchikumbura et al. 2014).

*Neopestalotiopsis* commonly occurs as a plant pathogen and together with *Pestalotiopsis* represents a fungal group known to produce a wide range of chemically novel metabolites (Maharachchikumbura et al. 2014). These species have been recovered from soil, polluted stream water, wood, paper, fabric, and wool (Guba 1961) and are associated with human and animal infections (Sutton 1999).

## Discussion

HTS methods are quick to conduct millions of chemical or pharmacological tests. The results of these experiments provide starting points for the understanding of biochemical processes in biology and for the detection of enzymatic activities (Reetz 2002). Among the available HTS methodologies, those carried out in 96-well microplates with modified substrates (fluorogenic probes) to reveal the enzymatic reaction by signals of the fluorophore products are the most common (Reymond 2006). These assays provide quantitative results and can be automated by the use of microplate readers for data acquisition.

Due to its successful application to human MAO-A and MAO-B enzymatic activities (Lu et al. 2008), 7-(3-aminopropoxy)coumarin (**1**) was selected as a fluorogenic probe for MAO enzymatic screening in microorganisms, based on our previous experience with whole-cell HTS experiments and analogous probes (Gonçalves and Marsaioli 2014; Lima et al. 2015; Mantovani et al. 2010). In this work, probe **1** was effective on selecting five fungi with possible MAO activity. However, by using this fluorogenic assay, it is not possible to differentiate between MAO and TA activities. As shown in Fig. 1, probe **1** produces, either by enzymatic oxidative deamination (MAO) or transamination (TA), the same aldehyde product (**3**), which undergoes spontaneous β-elimination releasing the fluorescent umbelliferyl anion (**4**).

Consequently, in order to overcome this issue, we performed biotransformation experiments using 2-methyl-6-alkylpiperidines as substrates to confirm the MAO activities observed in the five fungi selected by HTS. Biotransformation of these compounds must take place only by MAOs, producing the respective piperideines, as TAs do not catalyze reactions involving secondary and tertiary amines.

Using this approach, a monoamine oxidase was confirmed in *Neopestalotiopsis* sp. CBMAI 2030 (isolate F053), which transformed all evaluated piperidines and, on its best, deracemized 2-methyl-6-pentylpiperidine into (2*R*,6*S*)-2-methyl-6-pentylpiperidine in 38% conversion and 73% *ee* within 7 days. The piperideine enantiomeric excess was not accessed due to a lack of standards and enantiomeric discrimination methods. These results also attested the efficiency of probe **1** and the HTS technique in detecting new MAOs.

For years, 2-methyl-6-alkylpiperidines are the subject of research in our group. These alkaloids are also known as solenopsins because they are the main constituents of *Solenopsis* ant venom. The two stereocenters of the solenopsins allow the existence of four stereoisomers: *tran*s (*2R,6R* or *2S,6S*) and *cis* (*2R,6S* or *2S,6R*), and the ratio of these diastereoisomers in the venom composition varies between *Solenopsis* worker ants and queens (Pianaro et al. 2012).

Concerning biocatalytic synthesis, Reilly et al. (2014) developed a chemo-enzymatic process for the production of chiral 2,5-disubstituted pyrrolidines using transaminase and monoamine oxidase from *A. niger* (MAO-N), obtaining excellent enantioselectivity and diastereoselectivity. With the activity showed by *Neopestalotiopsis* sp. CBMAI 2030, this chemo-enzymatic process could be applied to give access to enantiomerically pure 2,6-disubstituted piperidines (solenopsins), shading light into the role of the absolute configuration and the ant communication inside the nests.

Fungal monoamine oxidase from *A. niger* and its variants are the main monoamine oxidases used as biocatalyst for the deracemization of secondary and tertiary amines (Carr et al. 2005; Dunsmore et al. 2006). The wild-type MAO-N is most active on simple straight-chain amines and shows poor activity with cyclic amines, which demanded several rounds of direct evolution to enhance its substrate acceptance (Carr et al. 2003). This characteristic creates the opportunity to search for new wild-type MAOs with distinct activity scope. Therefore, this report adds a new enzyme to the known MAO panel and the gene and heterologous expression of this novel MAO is under investigation.

## Electronic supplementary material

ESM 1 (PDF 744 kb)
