# Supplementary material for: Monoamine oxidase and transaminase screening: biotransformation of 2-methyl-6-alkylpiperidines by Neopestalotiopsis sp. CBMAI 2030
Source: Appl Microbiol Biotechnol. 2017 Jun 28;101(15):6061–70. doi: 10.1007/s00253-017-8389-z (PMC5522522; doi:10.1007/s00253-017-8389-z)
Supplement: Supplementary file 1 — (PDF 744 kb) [file 253_2017_8389_MOESM1_ESM.pdf]

Applied Microbiology and Biotechnology

Monoamine oxidase and transaminase screening: biotransformation of 2-methyl-6-alkylpiperidines by *Neopestalotiopsis* sp. CBMAI 2030

Jonas Henrique Costa[1], Bruna Zucoloto da Costa[1], Derlene Attili de Angelis[2], Anita Jocelyne Marsaioli[1*]

[1]Institute of Chemistry, State University of Campinas - UNICAMP, PO Box 6154, 13083-970, Campinas, SP, Brazil

[2] Division of Microbial Resources, Chemical, Biological and Agricultural Pluridisciplinary Research Center - CPQBA, State University of Campinas - UNICAMP, 13148-218, Campinas, SP, Brazil.

[*]anita@iqm.unicamp.br Phone: +55 19 35213098

Supplementary Material

Spectra

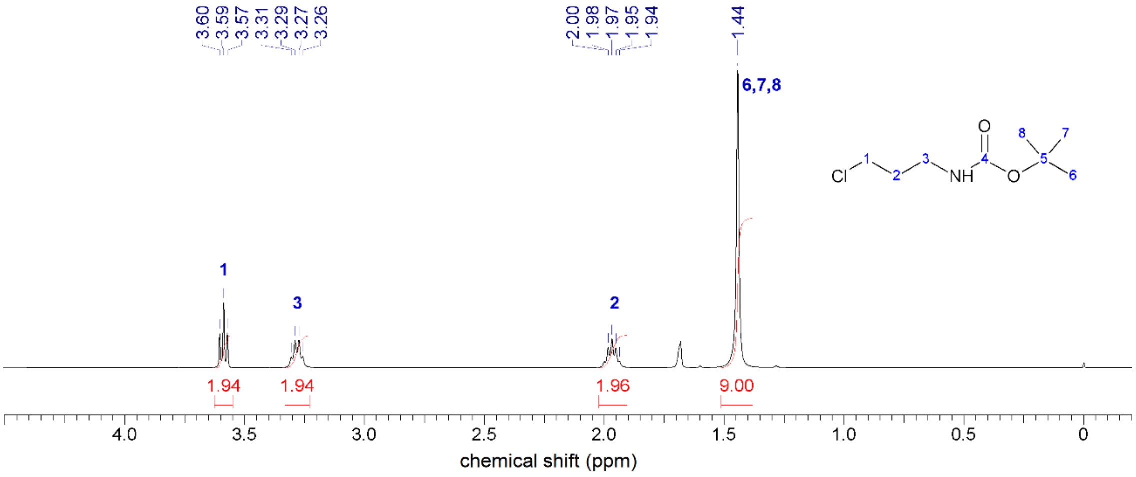

Fig. S1 ^{1}H NMR spectrum (400.18 MHz, $CDCl_3$) of **1c**

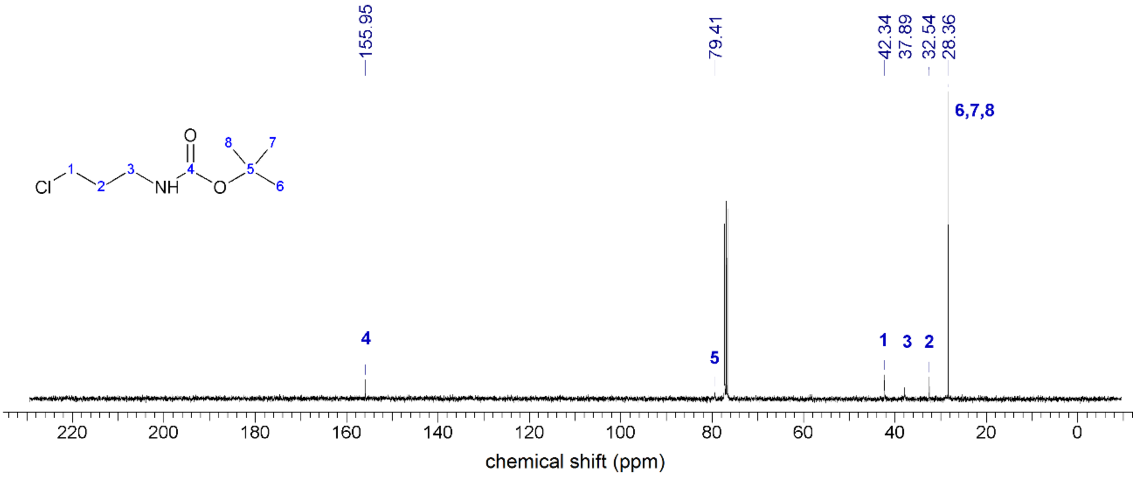

Fig. S2 ^{13}C NMR spectrum (100,63 MHz, $CDCl_3$) of **1c**

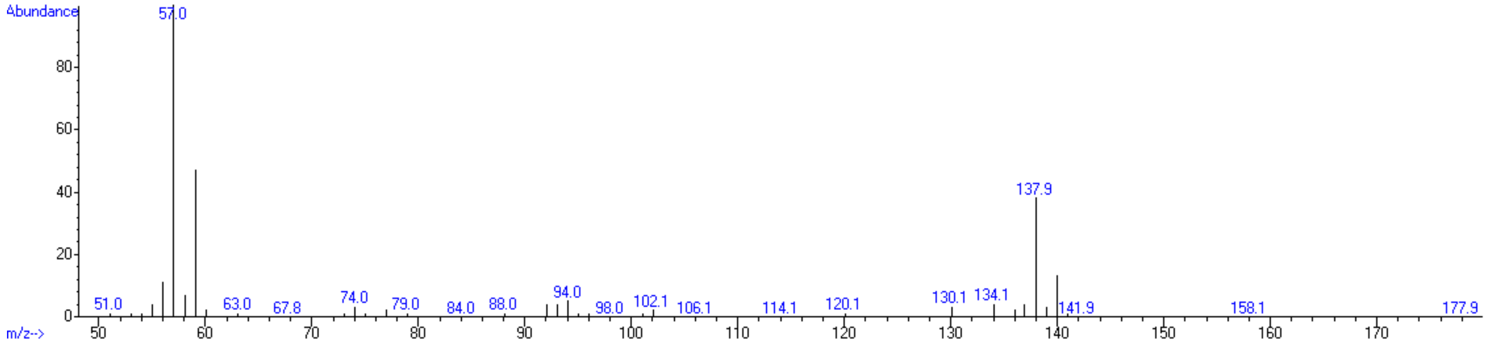

Fig. S3 EI-MS (70 eV) of **1c**

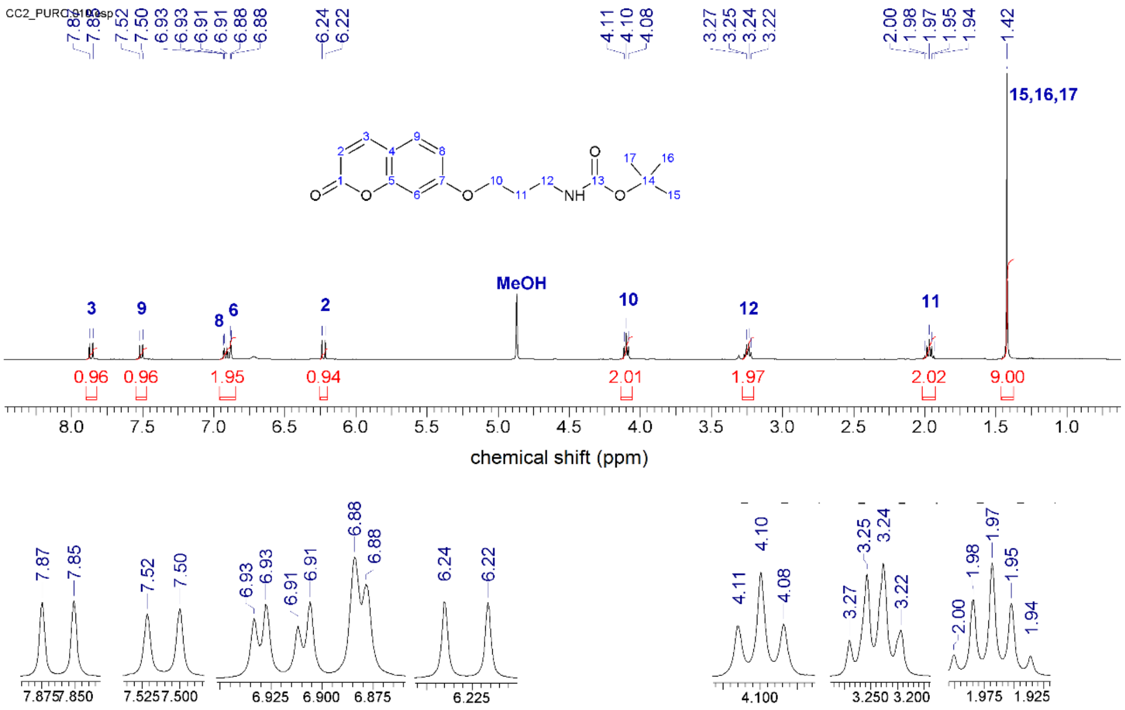

Fig. S4 ^{1}H NMR spectrum (400.18 MHz, $CDCl_3$) of **1d**

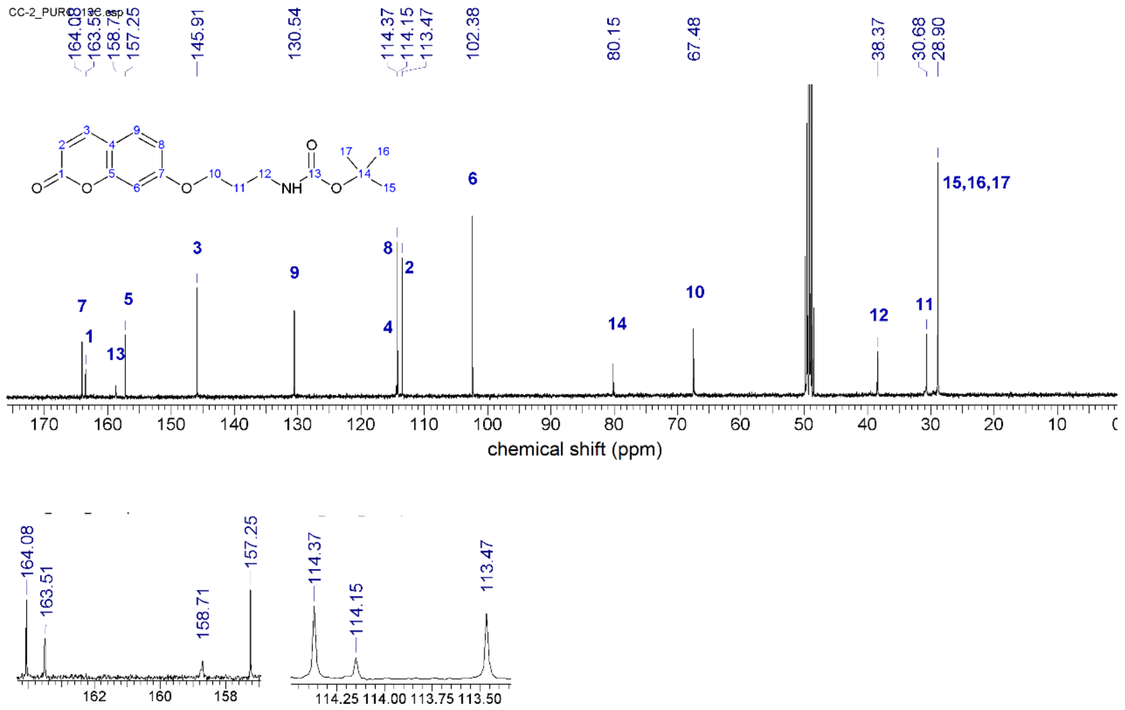

Fig. S5 ^{13}C NMR spectrum (100,63 MHz, $CDCl_3$) of **1d**

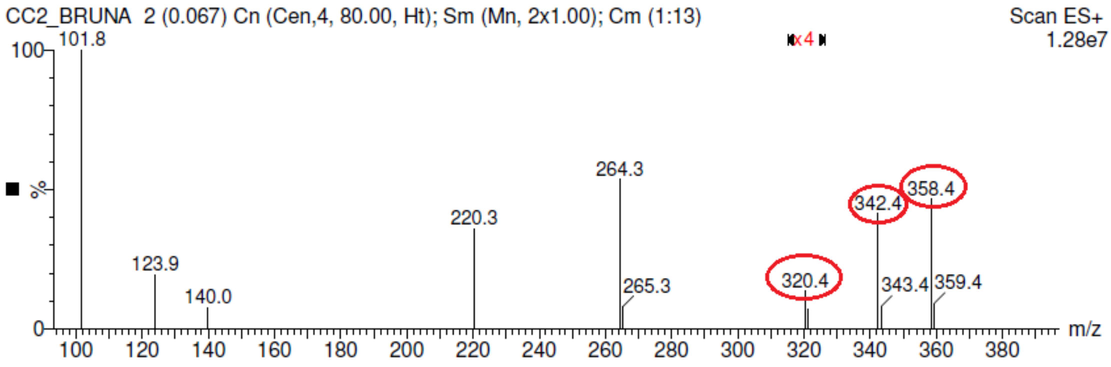

Fig. S6 ESI (+) mass spectrum of **1d**

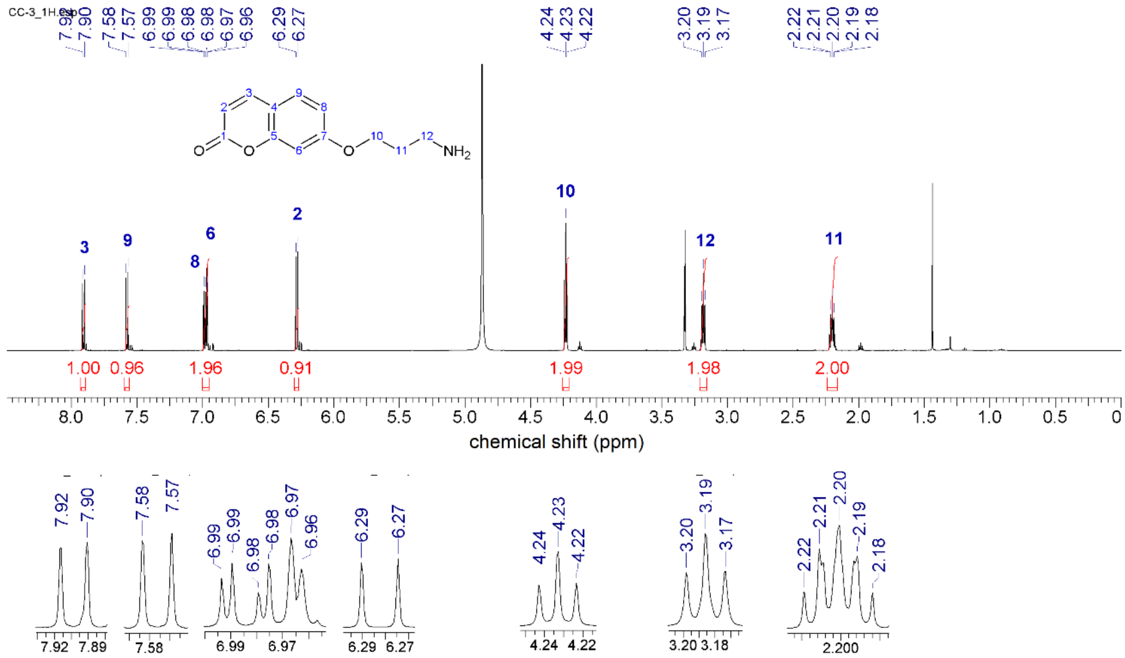

Fig. S7 ^{1}H NMR spectrum (400.18 MHz, CD_3OD) of **1**

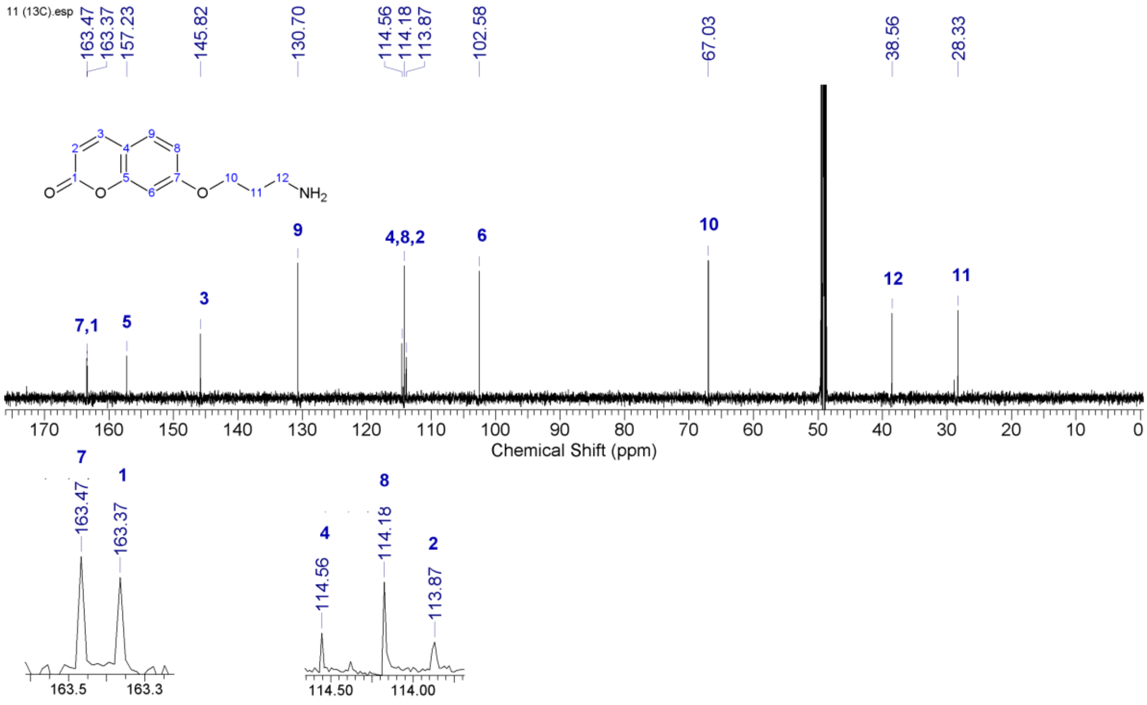

Fig. S8 ^{13}C NMR spectrum (150,91 MHz, CD_3OD) of **1**

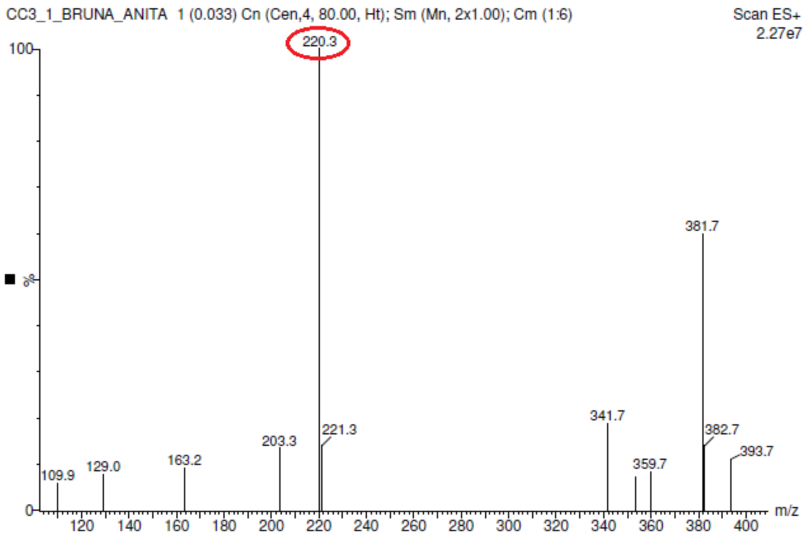

Fig. S9 ESI (+) mass spectrum of **1**

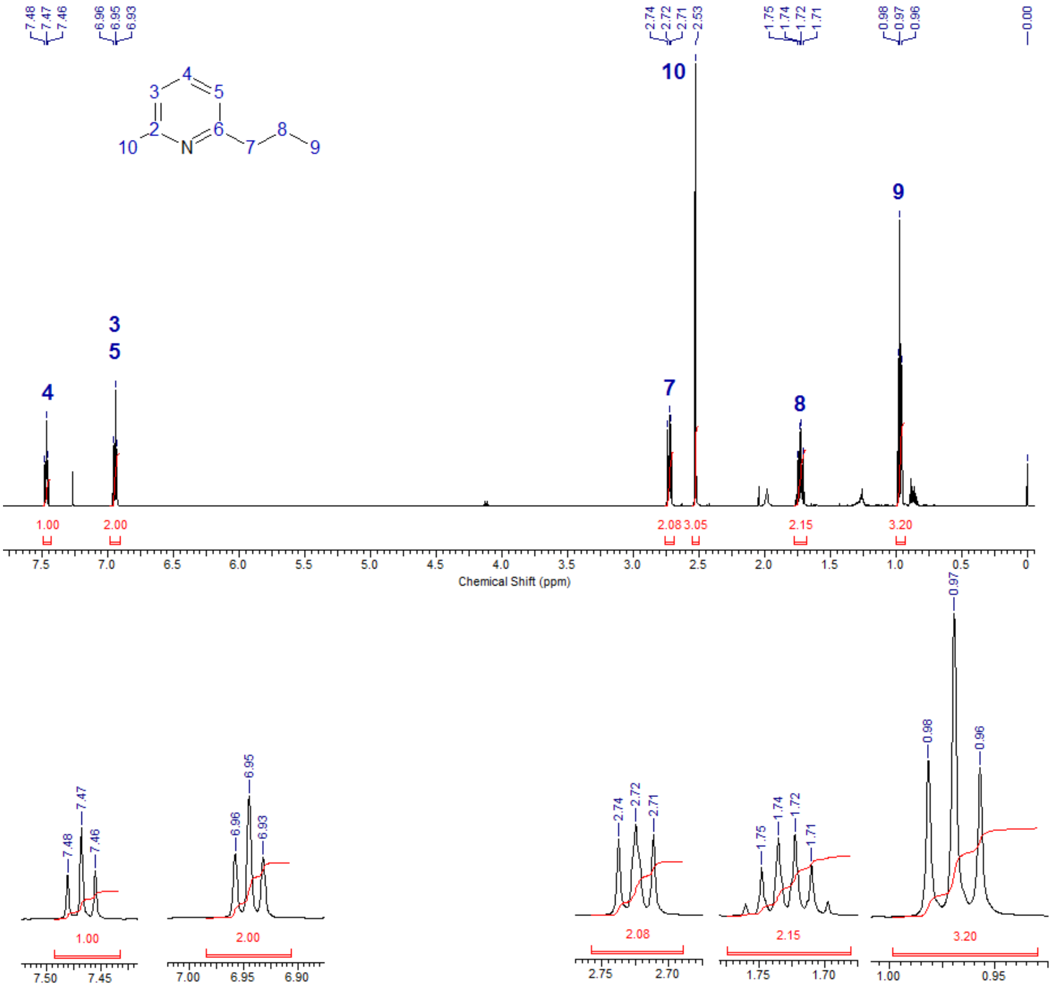

Fig. S10 ^{1}H NMR spectrum (600,17 MHz, $CDCl_3$) of **6b**

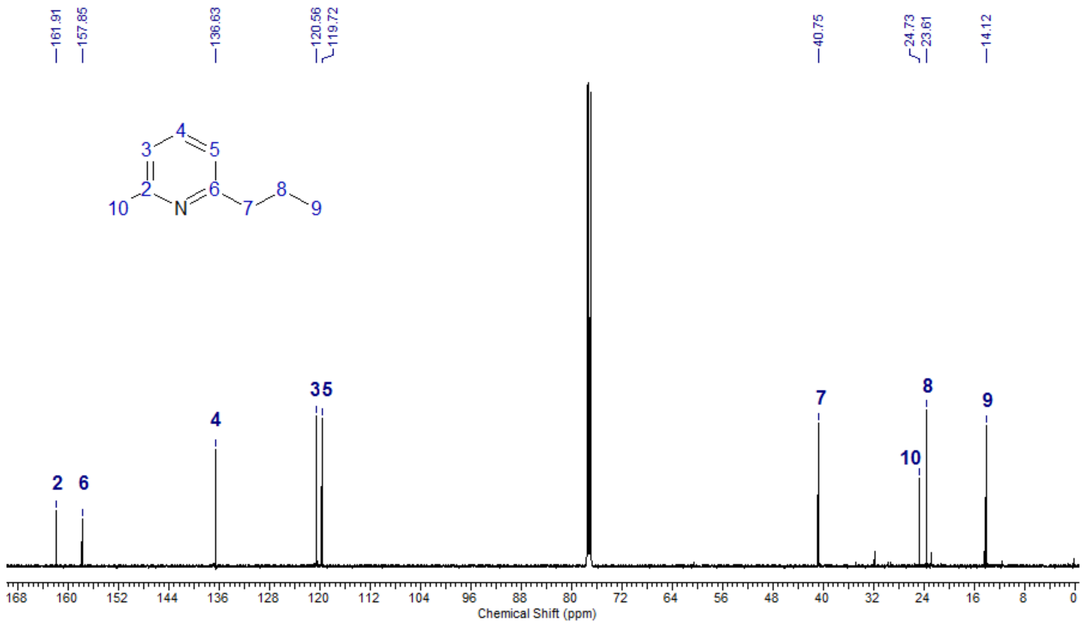

Fig. S11 ^{13}C NMR spectrum (150,92 MHz, $CDCl_3$) of **6b**

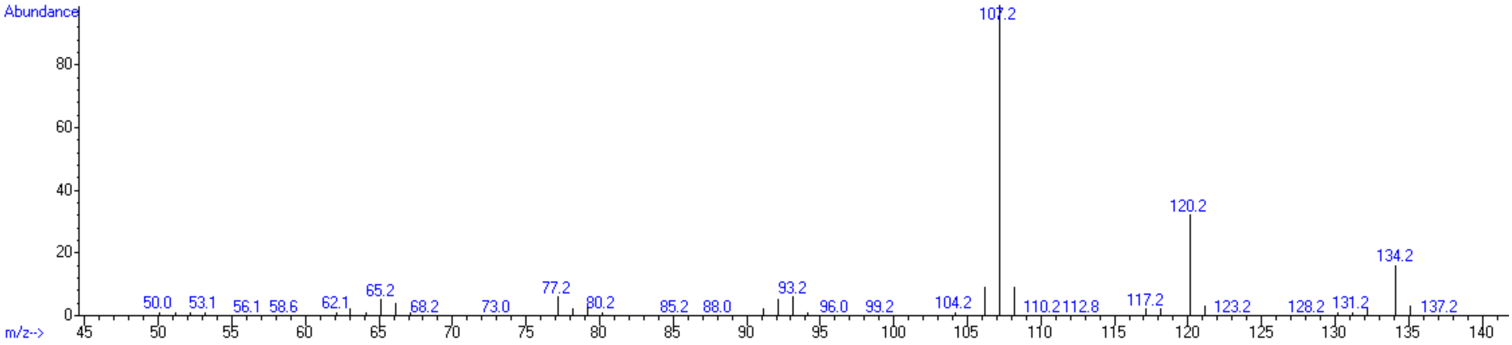

Fig. S12 EI-MS spectrum (70 eV) of **6b**

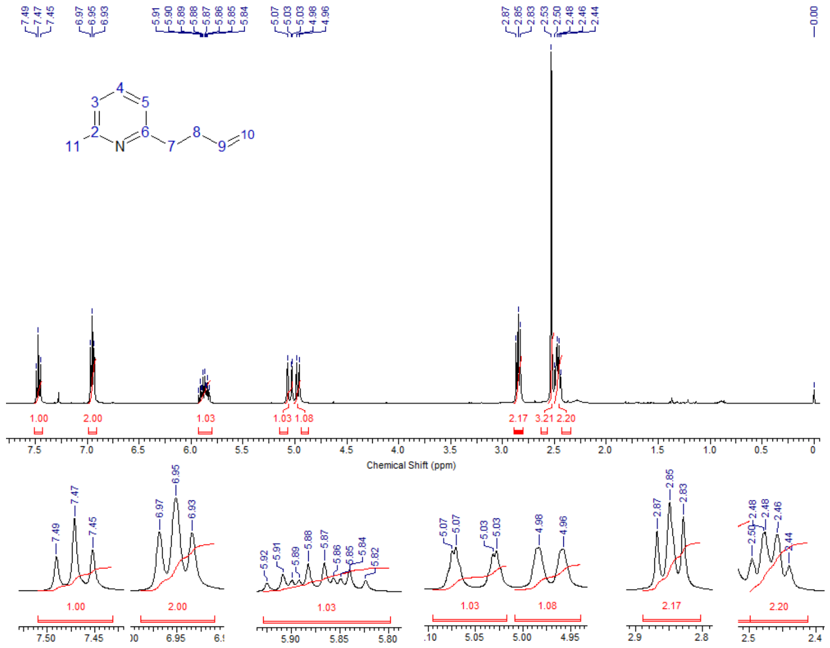

Fig. S13 ^{1}H NMR spectrum (400,18 MHz, $CDCl_3$) of **7b**

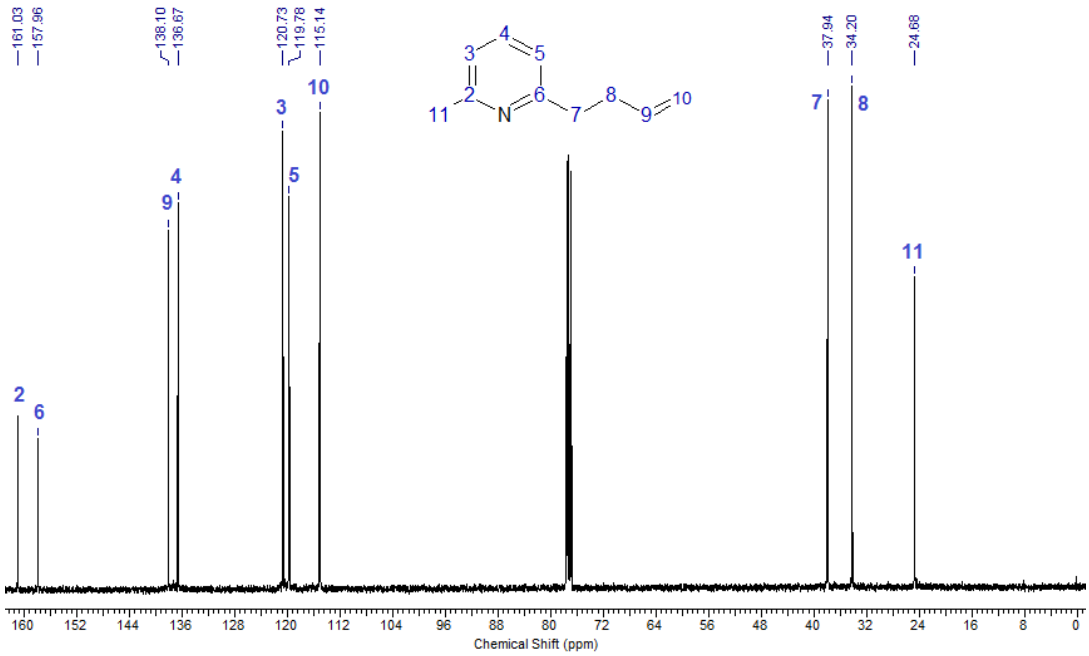

Fig. S14 ^{13}C NMR spectrum (100,63 MHz, $CDCl_3$) of **7b**

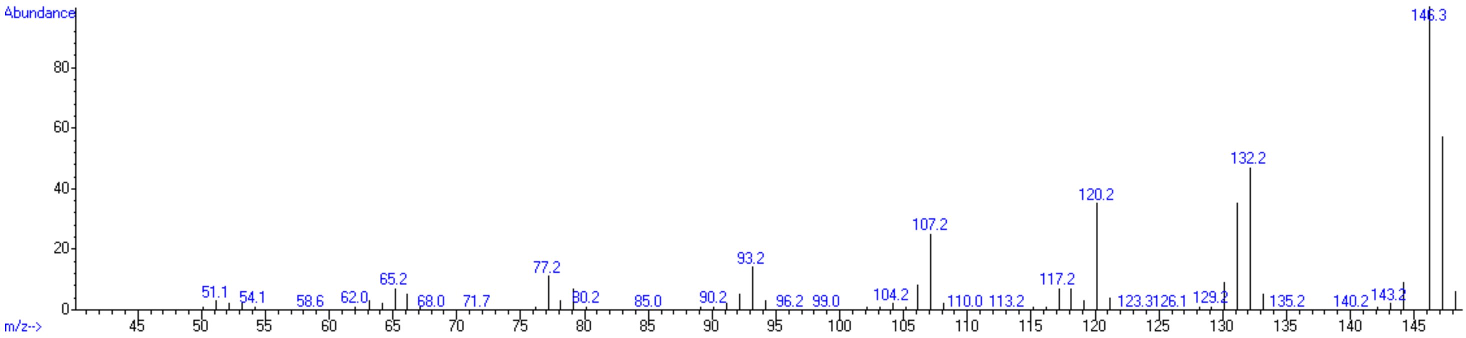

Fig. S15 EI mass spectrum (70 eV) of **7b**

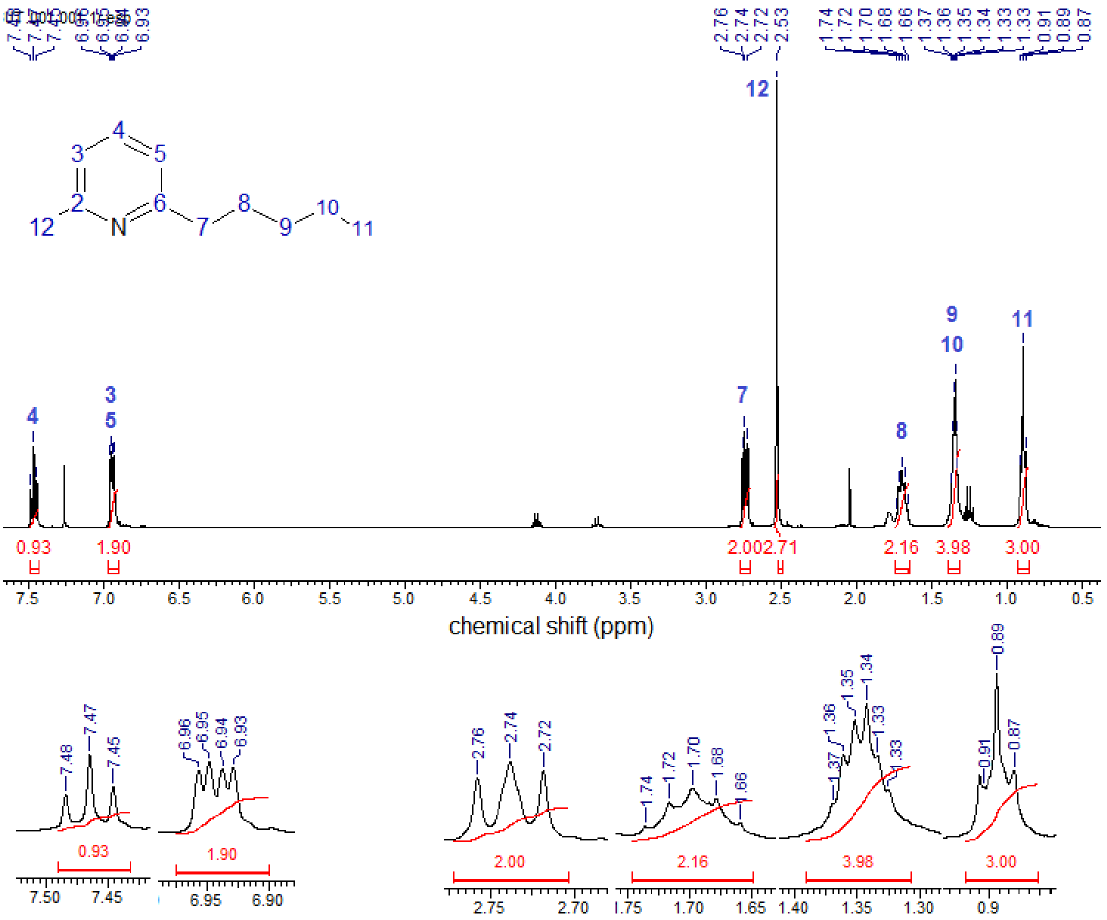

Fig. S16 ^{1}H NMR spectrum (400,18 MHz, $CDCl_3$) of **8b**

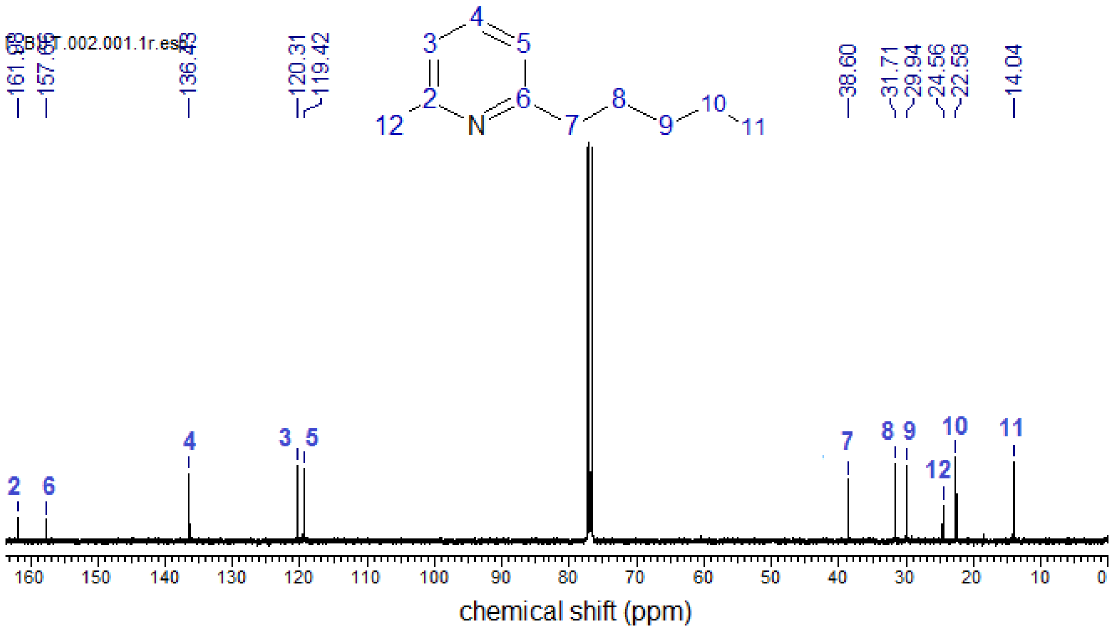

Fig. S17 ^{13}C NMR (100,63 MHz, $CDCl_3$) spectrum of **8b**

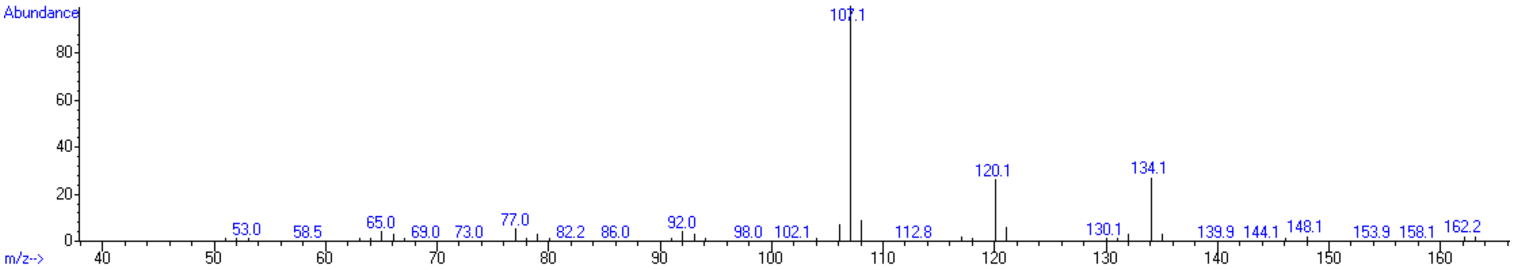

Fig. S18 EI mass spectrum (70 eV) of **8b**

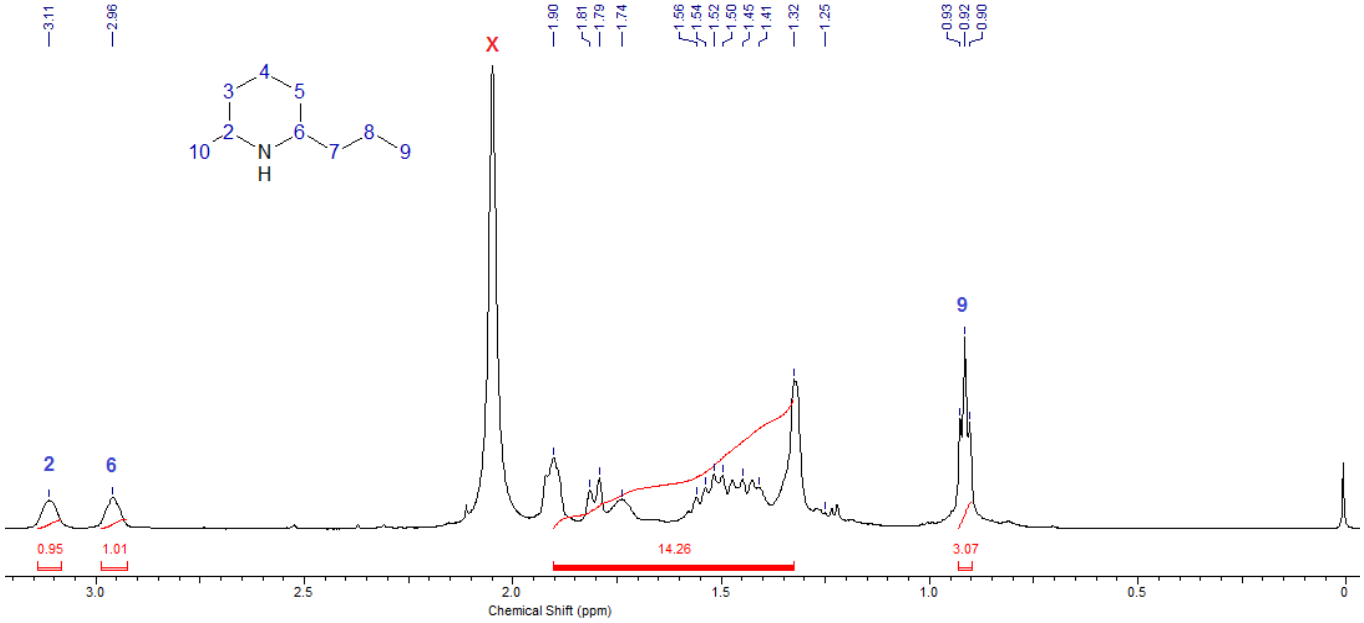

Fig. S19 ^{1}H NMR spectrum (600,17 MHz, $CDCl_3$) of **6**

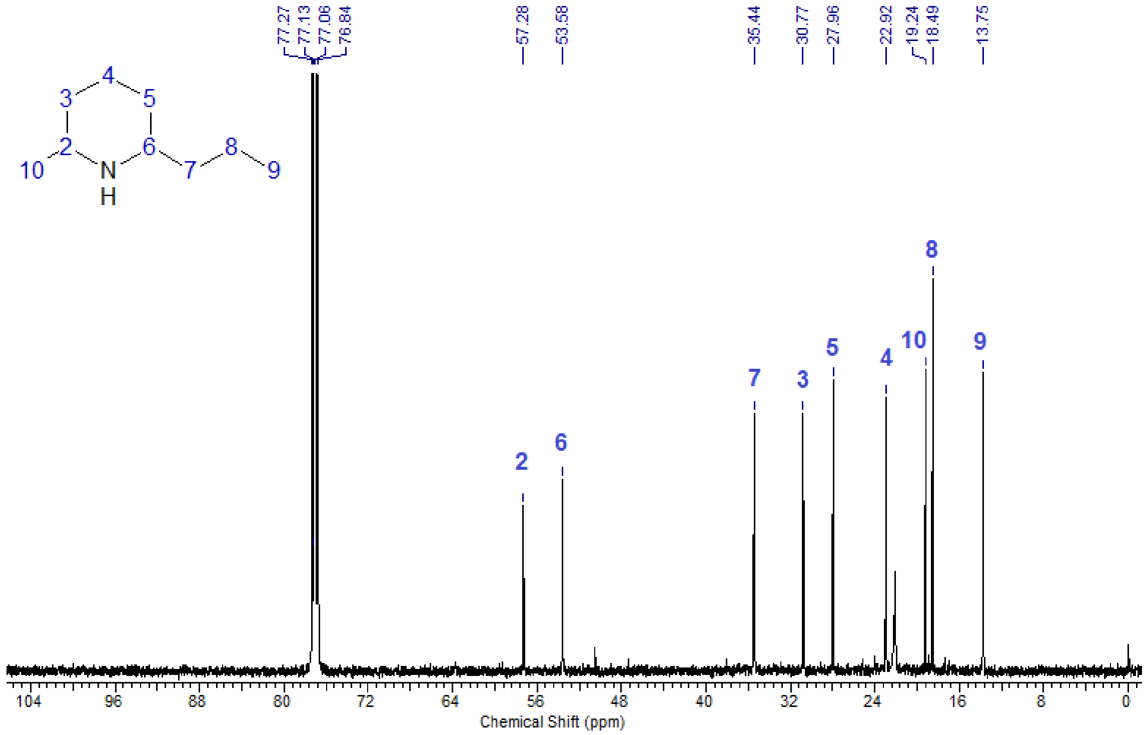

Fig. S20 ^{13}C NMR spectrum (150,93 MHz, $CDCl_3$) of **6**

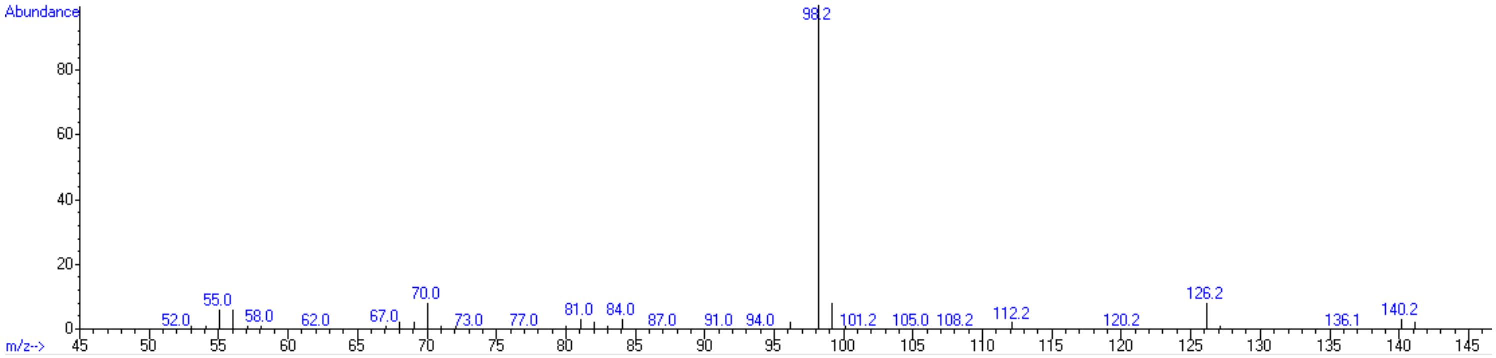

Fig. S21 EI mass spectrum (70 eV) of **6**

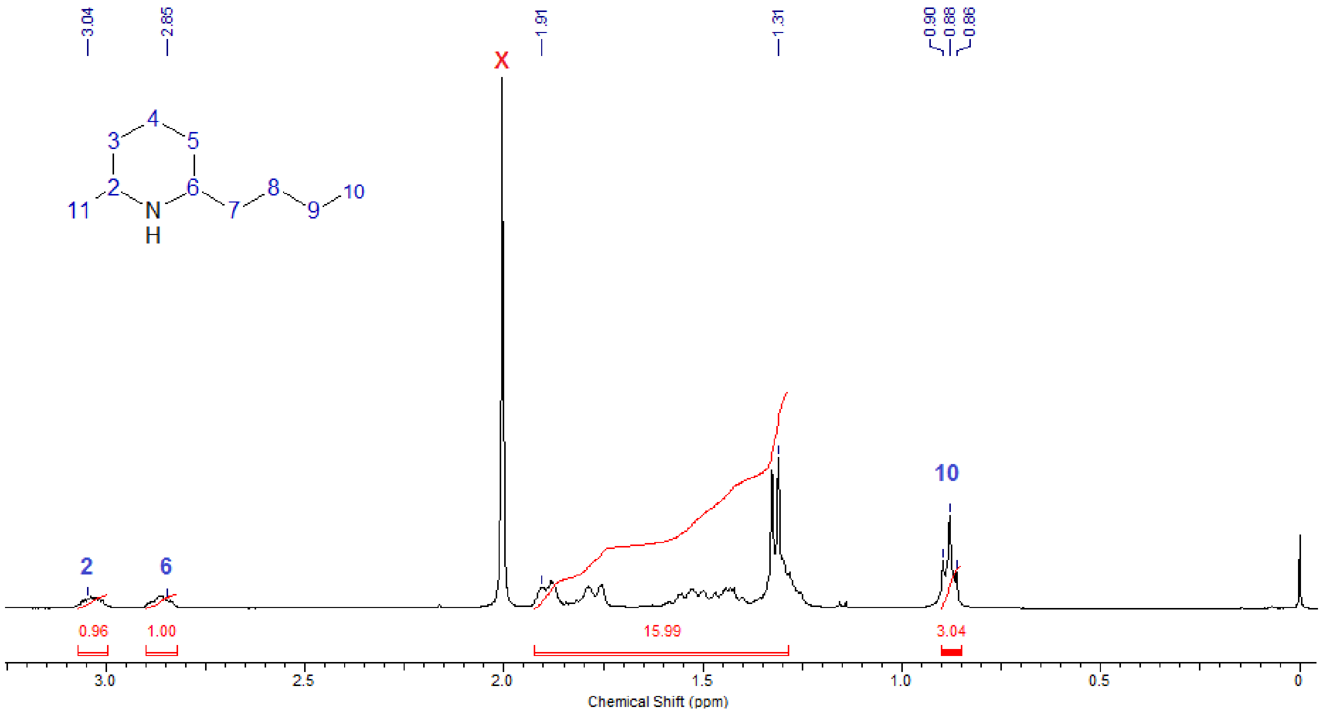

Fig. S22 ^{1}H NMR spectrum (400,18 MHz, $CDCl_3$) of **7**

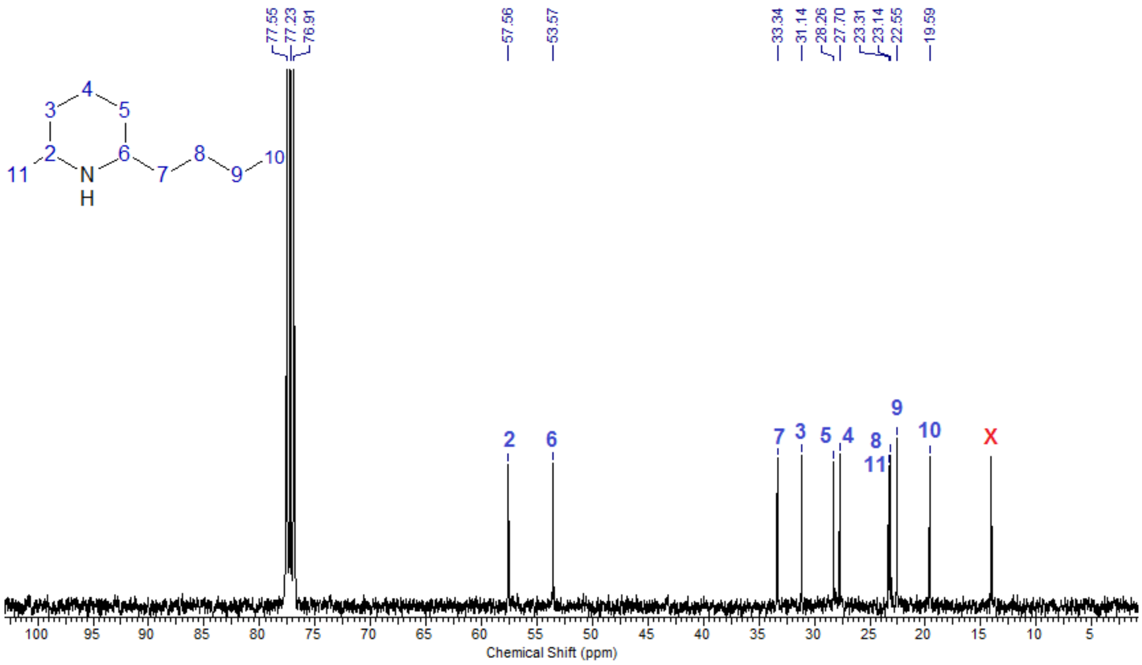

Fig. S23 ^{13}C NMR spectrum (100,63 MHz, $CDCl_3$) of **7**

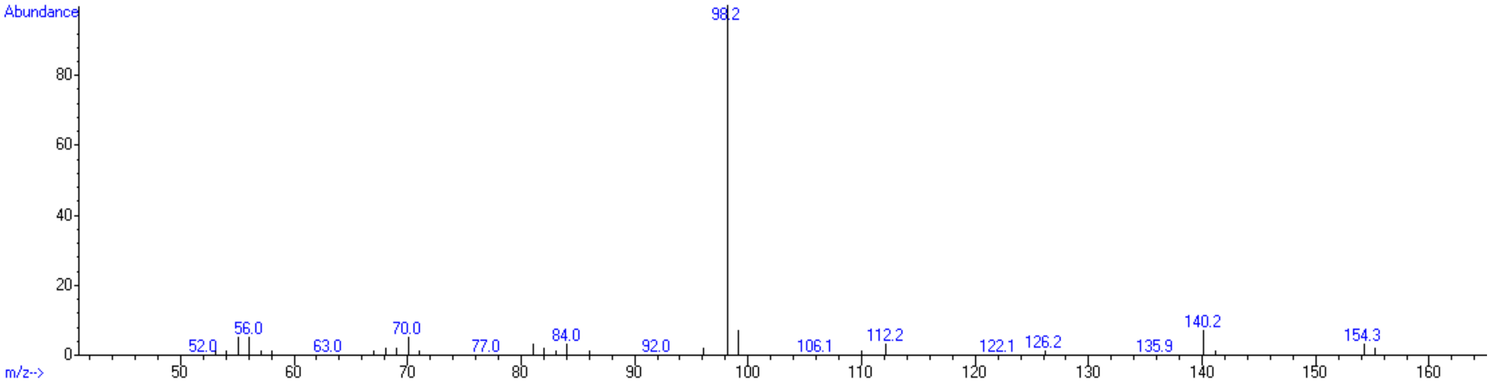

Fig. S24 EI mass spectrum (70 eV) of **7**

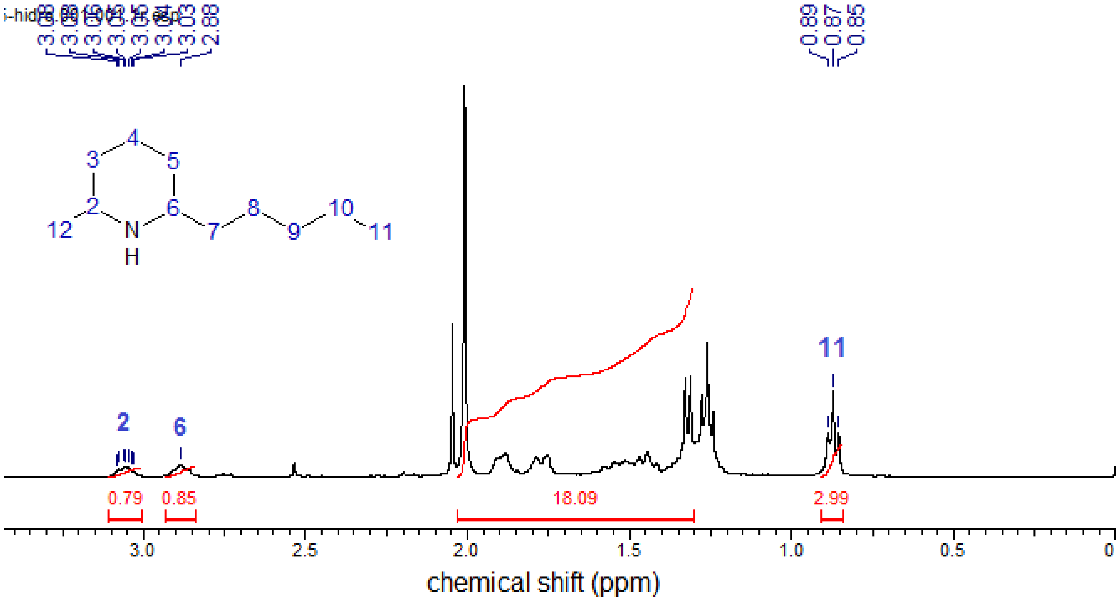

Fig. S25 ^{1}H NMR spectrum (400,18 MHz, $CDCl_3$) of **8**

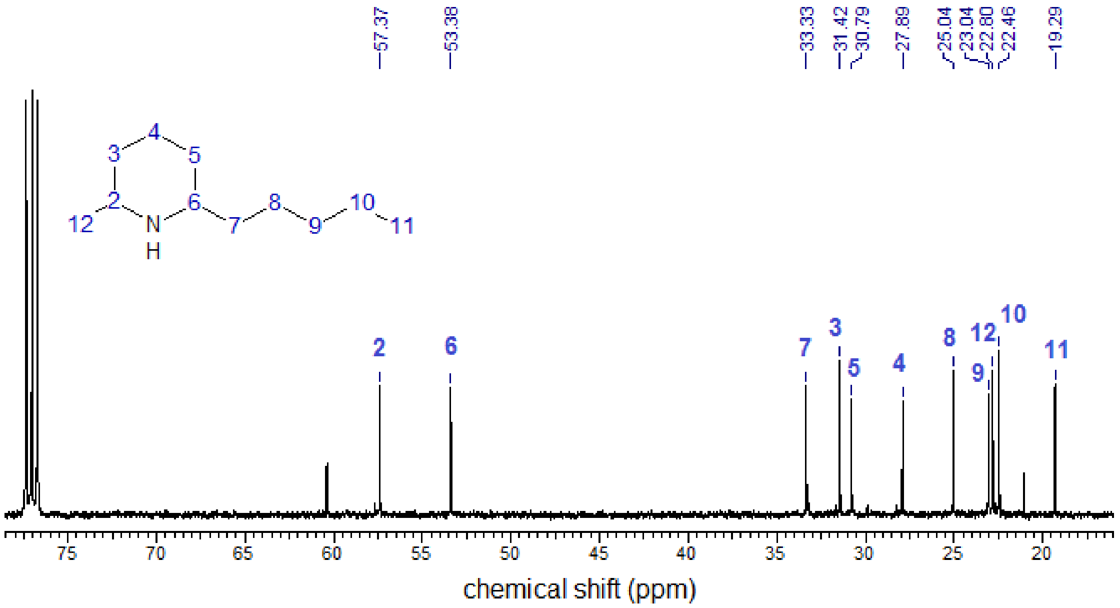

Fig. S26 ^{13}C NMR spectrum (100,63 MHz, $CDCl_3$) of **8**

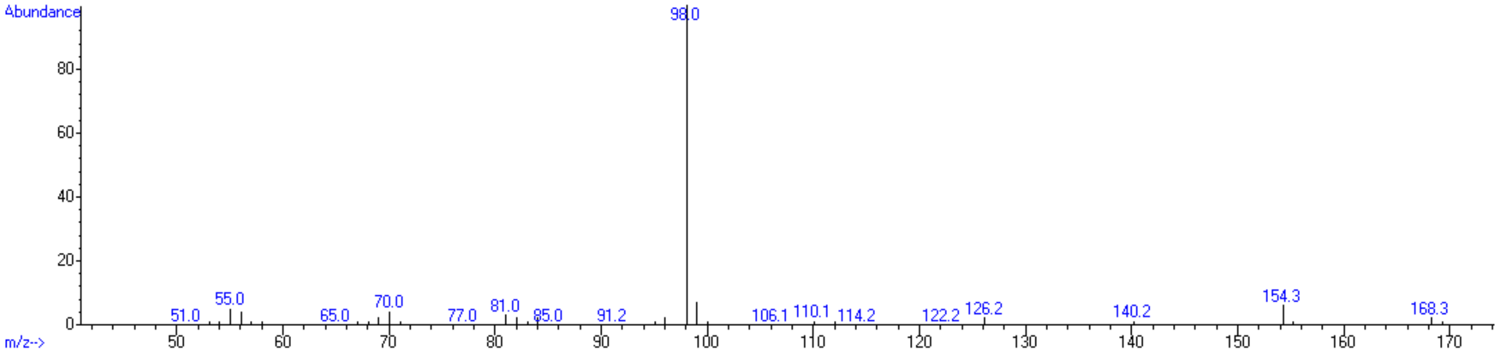

Fig. S27 EI mass spectrum (70 eV) of **8**

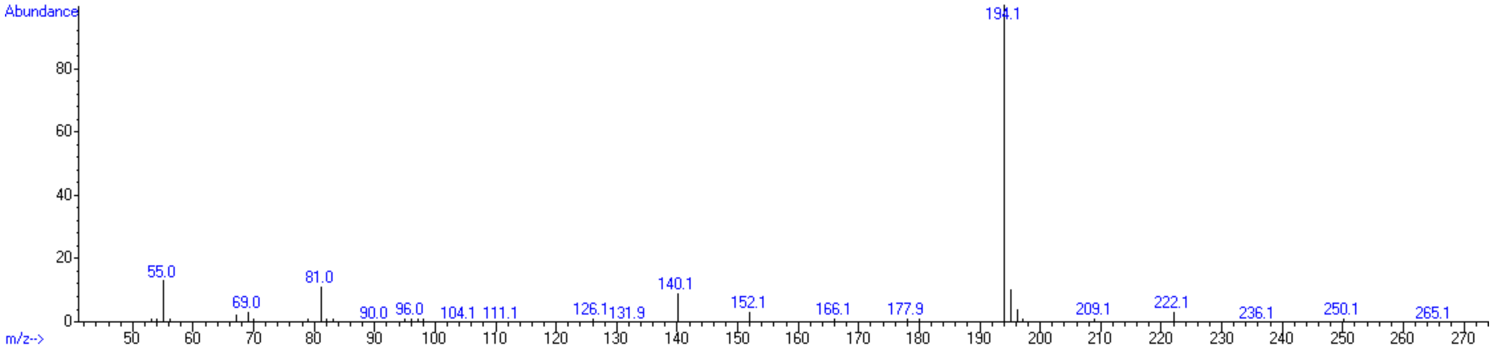

Fig. S28 EI mass spectrum (70 eV) of **11**
